# Association of cerebrovascular morphology with ischaemic stroke considering intracranial stenosis

**DOI:** 10.1093/braincomms/fcag037

**Published:** 2026-02-10

**Authors:** Eun Sol Cho, Jong-Un Choi, Ha-Na Song, In-Young Baek, Ji-Eun Lee, Hyun Kyung Kim, Wook Kim, Hwan-Ho Cho, Hyung Jun Kim, Jong-Won Chung, Oh Young Bang, Gyeong-Moon Kim, David S Liebeskind, Hyunjin Park, Woo-Keun Seo

**Affiliations:** Department of Neurology, Samsung Medical Center, Sungkyunkwan University School of Medicine, Seoul 06351, South Korea; Department of Neurology, Samsung Medical Center, Sungkyunkwan University School of Medicine, Seoul 06351, South Korea; Department of Digital Health, Samsung Advanced Institute for Health Sciences and Technology, Sungkyunkwan University, Seoul 06351, South Korea; Department of Neurology, Samsung Medical Center, Sungkyunkwan University School of Medicine, Seoul 06351, South Korea; Department of Neurology, Samsung Medical Center, Sungkyunkwan University School of Medicine, Seoul 06351, South Korea; Department of Neurology, Samsung Medical Center, Sungkyunkwan University School of Medicine, Seoul 06351, South Korea; Department of Neurology, Samsung Medical Center, Sungkyunkwan University School of Medicine, Seoul 06351, South Korea; Department of Radiology, Samsung Medical Center, Sungkyunkwan University, Seoul 06351, Korea; Department of Electronics Engineering, Incheon National University, Incheon 22012, South Korea; Department of Neurology, Samsung Medical Center, Sungkyunkwan University School of Medicine, Seoul 06351, South Korea; Department of Neurology, Samsung Medical Center, Sungkyunkwan University School of Medicine, Seoul 06351, South Korea; Department of Neurology, Samsung Medical Center, Sungkyunkwan University School of Medicine, Seoul 06351, South Korea; Department of Neurology, Samsung Medical Center, Sungkyunkwan University School of Medicine, Seoul 06351, South Korea; Department of Neurology, University of California Los Angeles, Los Angeles, CA 90095, USA; Department of Electrical and Computer Engineering, Sungkyunkwan University, Suwon 16419, South Korea; Center for Neuroscience Imaging Research, Institute for Basic Science, Suwon 16419, South Korea; Department of Neurology, Samsung Medical Center, Sungkyunkwan University School of Medicine, Seoul 06351, South Korea; Department of Digital Health, Samsung Advanced Institute for Health Sciences and Technology, Sungkyunkwan University, Seoul 06351, South Korea

**Keywords:** ischaemic stroke, intracranial atherosclerotic stenosis

## Abstract

Advances in imaging technologies and computational analyses have enabled the comprehensive evaluation of cerebrovascular morphology, thereby facilitating the identification of predictive indicators of cerebrovascular events. This study evaluated the association between the cerebral arterial morphological features and the incidence of ischaemic stroke while considering intracranial atherosclerotic stenosis and vascular distribution. This retrospective study analysed data from patients with acute ischaemic stroke or transient ischaemic attack between January 2017 and December 2020 who underwent time-of-flight magnetic resonance angiography at least twice over a minimum interval of 3 years. Data were analysed between September 2003 and November 2023. Arterial features, including baseline measurements and annual changes, were quantified across the entire cerebrovascular system using in-house vessel analysis software. Kaplan–Meier survival analysis and Cox proportional hazards regression were performed to assess the association between the arterial features and ischaemic stroke stratified according to the presence of intracranial atherosclerotic stenosis. Of the total study population of 462 patients (mean age 64.35 ± 13.22 years, 64.29% male; mean follow-up 5.28 ± 2.58 years, up to 10 years), 235 experienced acute ischaemic stroke during the follow-up period. Several global features associated with ischaemic stroke were identified. The baseline minimum–maximum diameter ratio and luminal circularity exhibited positive correlations with ischaemic stroke, whereas the baseline curvature exhibited a negative correlation. A chunk-based feature analysis, which accounted for the vascular distribution, was conducted to explore these associations. The baseline minimum–maximum diameter ratio exhibited positive correlations primarily in small arteries. In contrast, the baseline curvature exhibited negative correlations in specific regions, such as parts of the posterior circulation and the left middle cerebral artery. The subgroup analysis revealed differing patterns of association based on the presence of intracranial atherosclerotic stenosis. The correlation patterns observed in the total population were largely preserved in the group without stenosis, whereas no significant correlations were identified in the group with stenosis. These findings suggest that intracranial atherosclerotic stenosis may obscure these relationships. Thus, the present study highlights the importance of integrating whole-brain vascular morphology analysis with the stratification of intracranial atherosclerotic stenosis to understand the mechanisms of ischaemic stroke. These findings provide insights into potential predictive metrics that will contribute to the development of advanced models for personalized stroke prevention and management.

## Teaser

Cho *et al*. report that specific morphological features of cerebral vessels—luminal shape and curvature—are associated with ischaemic stroke, with analyses stratified by intracranial atherosclerotic stenosis. Findings support the development of more accurate, individualized risk assessment models to improve prevention and patient-specific management.

## Graphical Abstract

**Graphical Abstract:**
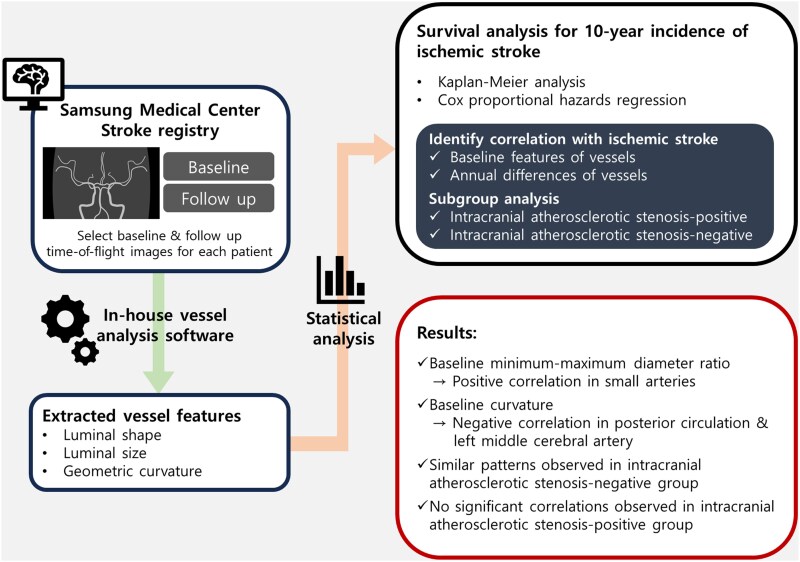

## Introduction

The rising global incidence of ischaemic stroke and its significant role as a leading cause of death highlight the clinical importance of its prediction and prevention.^1,2^ Although established vascular risk factors are important determinant for stroke,^3,4^ neuroimages can provide additional insights into its pathophysiology and outcome. Recent advances in imaging technologies for visualizing cerebrovascular morphology along with computational analysis have enabled comprehensive analyses of the arterial structure.^5^ Previous studies of brain images often focused on individual vascular parameters, such as luminal stenosis.^6-8^ While luminal stenosis is associated with stroke risk, relying exclusively on this metric may have resulted in the potential contributions of arterial morphology to stroke development being overlooked. Moreover, the relationship between whole-brain arterial features and ischaemic stroke remains largely unexplored as most analyses have been confined to select arterial regions.^9^ Thus, a more comprehensive approach must be adopted to understand the structural characteristics of cerebral arteries and their potential role in stroke risk. In particular, severity of intracranial atherosclerotic stenosis (ICAS) has been linked with the recurrence of stroke. Thus, ICAS must be accounted for when evaluating the association between the arterial morphological features and the incidence of ischaemic stroke.^7,8^

This study performed a quantitative analysis of the morphological features of cerebrovascular arteries at the whole-brain level. Using vessel analysis software, we explored the association between the arterial features and ischaemic stroke across the entire cerebrovascular system, providing deeper insights into their potential role in the pathophysiology of ischaemic stroke.

## Materials and methods

### Data of the study population

The data of patients who experienced acute ischaemic stroke or transient ischaemic attack (TIA) between January 2017 and December 2020 were extracted from the Samsung Medical Center (SMC) stroke registry and retrospectively analysed. Patients aged ≥19 years admitted to the neurology department of SMC within 7 days of symptom onset for acute ischaemic stroke or TIA included in the registry who provided informed consent after understanding the purpose of the study were eligible for inclusion.

The following inclusion criteria were applied to screen the patients included in the SMC stroke registry: (i) patients who had undergone magnetic resonance angiography (MRA) imaging, including TOF, at least twice with a minimum interval of 3 years between the imaging studies; (ii) patients who successfully underwent vessel extraction and morphological feature analysis using the in-house vessel analysis software program and (iii) patients with no history of intracerebral and subarachnoid haemorrhage or unruptured aneurysms.

The baseline was defined as the time of registry enrollment or, for patients with prior TOF imaging, the earliest available TOF scan before enrollment. The follow-up period was up to 10 years from the baseline TOF scan. This study was approved by the Institutional Review Board (IRB No. 2023-12-067).

### MRA image acquisition

Baseline and follow-up MRA were performed using 3.0 T MRI systems, predominantly manufactured by Philips Medical Systems/Philips Healthcare.

At baseline, the most common acquisition parameters were an echo time of 3.45 ms (89.6%), a repetition time of 25 ms (85.9%), a flip angle of 20° (94.2%), a field of view of 250 × 250 mm (85.5%), a voxel matrix of 880 × 880 × 380 mm³ (43.5%), an acquisition time of 352 s (33.6%) and a pixel bandwidth of 124 (79.2%).

At follow-up, acquisition parameters were largely consistent with those at baseline, with the most frequent settings including an echo time of 3.45 ms (90.5%), a repetition time of 25 ms (80.5%), a flip angle of 20° (90.3%), a field of view of 250 × 250 mm (87.5%), a voxel matrix of 880 × 880 × 380 mm³ (80.1%), an acquisition time of 352 s (31.0%) and a pixel bandwidth of 124 (79.2%) (Supplementary Table 1).

### Measurement of the morphological features of cerebral arteries

TOF images in the Digital Imaging and Communications in Medicine (DICOM) format were utilized as the raw input data for vessel extraction by the in-house vessel analysis software program. Prior to vessel modeling, anisotropic voxels along the z-axis were resampled to an isotropic voxel scale using interpolation, thereby minimizing potential effects of slice thickness or partition differences across scans. A vascular map was constructed by the software using a segmentation-stacking method analysing the extracted 3D cerebral arterial image through the identification of ‘spots.’ The fundamental units are represented as cubic cells present along the arterial centerline at intervals of 0.2801 mm. These spots were aggregated into larger units known as ‘segments’ based on their morphological characteristics.^5^ The segments were further reconstructed into 62 ‘branches’ using feature vector extraction algorithms and subsequently organized into 20 ‘chunks’. Supplementary Fig. 1 presents the vascular distribution of each chunk. The arterial features extracted using the in-house vessel analysis software program included the following metrics: the minimum–maximum diameter ratio (MMR) and luminal circularity (LC), classified as luminal shape metrics; area, perimeter, maximal diameter (MaxD), minimal diameter (MinD) and maximal inscribed sphere radius (MISR), categorized as luminal size metrics; and geometric curvature metrics (Supplementary Table 2). These features were categorized based on their characteristics and correlations (Supplementary Fig. 2). The method of evaluating the arterial features using the in-house vessel analysis software program has been described in a previous study.^5^

The morphological features of the vessels extracted using two different methods at two analysis scales were analysed in the present study. First, the feature data from the arterial segments were averaged across the entire intracranial region to obtain the global features, providing a representative value for all cerebral arteries. Second, the ‘chunk feature’ was obtained from 20 individual chunks for each patient to obtain regional data based on the vascular distribution. The aforementioned feature data including baseline data from the images captured at the time of ischaemic stroke occurrence and annual differences, which were calculated by dividing the change between the baseline and follow-up data by the follow-up period (in years), were obtained for all patients. The baseline data of each feature were denoted with the subscript ‘base’ as in feature*_base_* in the present study, whereas the data representing the annual difference of each feature were denoted with the subscript ‘diff’, as in feature*_diff_*.

### Measurement of intracranial stenosis

TOF imaging was used to evaluate ICAS in the following arterial segments: both internal carotid arteries (ICA), both M1-M2 segments, both A1-A2 segments, the basilar artery, both V4 segments and both P1–P3 segments. The stenosis percentages were assessed using the North American Symptomatic Carotid Endarterectomy Trial (NASCET) method for ICA and the Warfarin-Aspirin Symptomatic Intracranial Disease (WASID) method for other intracranial cerebral arteries.^10,11^ Patients with stenosis of ≥50% in ≥1 arterial segments were classified as the ICAS-positive group; the remaining patients were classified as ICAS-negative.

### Statistical analysis

Data were analysed from medical records covering the period between September 2003 and November 2023, reflecting the retrospective collection of clinical and imaging data based on the timing of each patient’s baseline MRA. For demographic characteristics, continuous variables are presented as mean ± standard deviation (SD), whereas categorical variables are presented as frequencies and percentages. The distribution of each global feature at baseline and follow-up was calculated as the mean ± SD. Intergroup differences were assessed using a *t*-test with *P*-values.

The mean ± SD of the global features and those of chunk features were calculated and compared between the ICAS-positive and ICAS-negative groups to assess the effect of stenosis on the arterial features. The statistical significance of the differences was assessed using t-tests based on *P*-values.

The Kaplan–Meier analysis was used to examine the association between global features and ischaemic stroke. The interval between the baseline and follow-up TOF scans was defined as the observation period. The occurrence of acute ischaemic stroke within 10 years of the baseline TOF scan was considered an event. The patients were stratified into high- and low-value groups based on the median value of the global features. The survival curves for the two groups were compared to assess their association with ischaemic stroke. The log-rank test was used to determine the statistical significance.

The clinical profiles, including age, sex, body mass index (BMI), hypertension, diabetes mellitus, dyslipidemia, current smoking, coronary artery disease, and atrial fibrillation, were analysed to further investigate the clinical characteristics of the high- and low-value groups with baseline global features. Continuous variables, such as age and BMI, are presented as the mean ± SD, and intergroup differences were assessed using t-tests. Categorical variables are presented as counts (percentages), and intergroup differences were assessed using the chi-square test to determine the statistical significance. Relevant adjustment factors were identified based on the results of this analysis and applied to a subsequent Cox proportional hazard regression.

The z-score was used to standardize each global feature. The hazard ratio (HR) for each feature in relation to ischaemic stroke was estimated using Cox proportional hazards regression after adjusting for age and sex. The Wald test was used to evaluate the statistical significance of the regression coefficients. The vascular distribution patterns of the association between arterial features and ischaemic stroke were explored using the Cox proportional hazards analysis for each chunk. Subgroup analysis was performed by dividing the patients into ICAS-positive and ICAS-negative groups, considering the effect of the severity of stenosis on arterial features.

## Results

### Baseline characteristics of the study population

**Figure 1:**
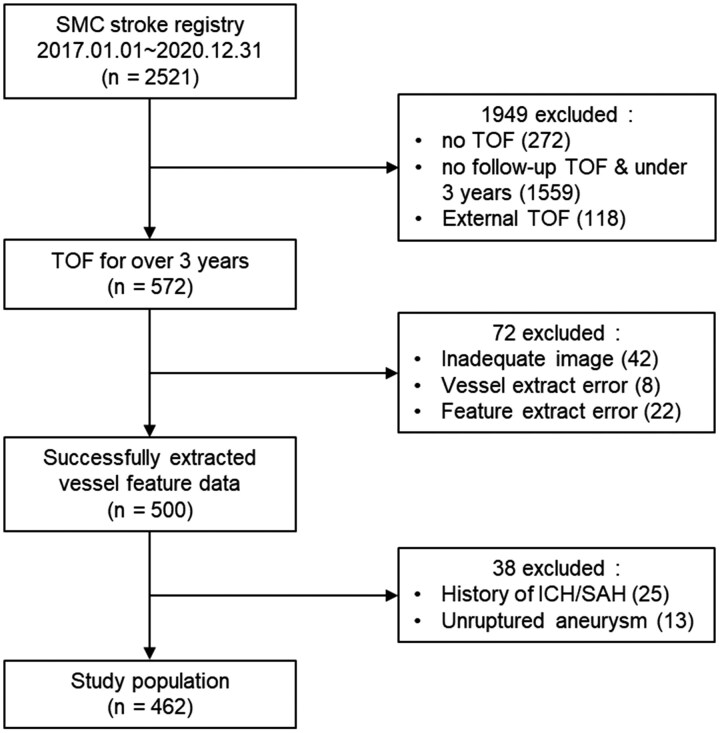
**Selection of the study population.** SMC, Samsung Medical Center; TOF, time-of-flight; MRA, magnetic resonance angiography; ICH, intracerebral haemorrhage; SAH, subarachnoid haemorrhage.

A total of 2521 patients were screened from the SMC stroke registry between January 2017 and December 2020. This population included 572 patients who underwent follow-up MRAs at intervals of >3 years. Among these 2521 patients, 1949 patients were excluded owing to the MRA studies being performed at intervals of <3 years, absence of TOF images, lack of follow-up MRA studies, or cases wherein TOF images were acquired externally. Furthermore, 42 patients with inappropriate MRA images, 8 with errors during the vessel extraction process using the in-house vessel analysis software program, and 22 with errors in the extraction of morphological features were excluded. Lastly, patients with a history of intracerebral haemorrhage (ICH), subarachnoid haemorrhage (SAH), or unruptured aneurysms were excluded. Thus, the final sample included 462 patients (Fig. 1).

Among the 462 patients included in the study (mean age: 64.35 ± 13.22 years, 64.29% males), 271 (58.66%) had baseline TOF imaging performed at the time of an acute ischaemic stroke or TIA, and 235 individuals (50.87%) experienced acute ischaemic stroke within 10 years. The mean interval between the baseline and follow-up TOF was 6.27 ± 3.43 years, and the mean follow-up period was 5.28 ± 2.58 years. The ICAS-positive and ICAS-negative groups comprised 245 (53.03%) and 217 (46.97%) individuals, respectively.

The distribution of the patients at the time of the ischaemic stroke event according to the Trial of Org 10172 in Acute Stroke Treatment (TOAST) classification was as follows: 70 (15.15%) patients with large artery atherosclerosis, 54 (11.69%) with cardioembolism, 52 (11.26%) with small artery occlusion, 21 (4.55%) with other uncommon causes, and 38 (8.23%) with undetermined causes (Table 1).

**Table 1:** Demographic characteristics of the 462 patients in the study population

Features	Values
Age, year	64.35 ± 13.22
Height, cm	162.91 ± 9.14
Weight, kg	65.48 ± 12.45
Body mass index (kg/m^2^)	24.59 ± 3.76
Sex, male	297 (64.29)
Interval between images, year	6.27 ± 3.43
Risk factors of ischaemic stroke	
Hypertension	315 (68.18)
Diabetes mellitus	136 (29.44)
Dyslipidemia	204 (44.16)
Current smoker	93 (20.13)
Atrial fibrillation	56 (12.12)
Coronary artery disease	66 (14.29)
Intracranial stenosis	
ICAS-positive	245 (53.03)
ICAS-negative	217 (46.97)
Previous TIA/ischaemic stroke	354 (76.62)
Follow-up period	5.28 ± 2.58
TOAST classification	
Baseline acute ischaemic stroke/transient ischemic attack	271 (58.66)
Transient ischaemic attack	43 (9.31)
Large artery atherosclerosis	74 (16.02)
Cardioembolism	52 (11.26)
Small artery occlusion	37 (8.01)
Other determined aetiology	30 (6.50)
Undetermined aetiology	35 (7.58)
Acute ischaemic stroke during the follow-up period	235 (50.87)
Large artery atherosclerosis	70 (15.15)
Cardioembolism	54 (11.69)
Small artery occlusion	52 (11.26)
Other determined aetiology	21 (4.55)
Undetermined aetiology	38 (8.23)

ICAS, intracranial atherosclerotic stenosis; TOAST, Trial of Org 10172 in Acute Stroke Treatment.

Continuous variables are expressed as mean ± standard deviation, whereas categorical variables are presented as numeric values (percentages).

Table 2 presents the distribution of the values of the extracted global features, with the following cases exhibiting significant differences between the baseline and follow-up groups: the luminal shape metrics, including MMR (baseline: 0.829 ± 0.025; follow-up: 0.840 ± 0.018; *P* < 0.001) and LC (baseline: 0.963 ± 0.008; follow-up: 0.965 ± 0.006; *P* < 0.001), and the luminal size metrics, including MinD (baseline: 2.610 ± 0.250; follow-up: 2.661 ± 0.245; *P* = 0.002) and MISR (baseline: 1.294 ± 0.115; follow-up: 1.319 ± 0.113; *P* = 0.001). In contrast, no significant differences were observed in terms of the geometric curvature.

**Table 2:** Comparison between the global features of the baseline and follow-up images of the 462 patients

Global features		Baseline	Follow-up	*P*-value
Luminal shape	Minimum–maximum diameter ratio	0.829 ± 0.025	0.840 ± 0.018	<0.001
	Luminal circularity	0.963 ± 0.008	0.965 ± 0.006	<0.001
Luminal size	Area	7.673 ± 1.543	7.840 ± 1.461	0.092
	Perimeter	9.308 ± 0.982	9.379 ± 0.852	0.241
	Maximum diameter	3.160 ± 0.344	3.174 ± 0.290	0.484
	Minimum diameter	2.610 ± 0.250	2.661 ± 0.245	0.002
	Maximum inscribed sphere radius	1.294 ± 0.115	1.319 ± 0.113	0.001
Geometric curvature	Curvature	0.298 ± 0.274	0.283 ± 0.020	0.264

### Patterns of arterial features according to intracranial stenosis

No statistically significant differences were observed between the ICAS-positive and ICAS-negative groups in terms of the mean global feature values (Supplementary Table 3).

A chunk-based analysis of the arterial features revealed several significant findings (*P* < 0.05). Notably, the MMR values of both internal carotid artery (ICA), both basal middle cerebral artery (MCA), basilar artery (BA), and right basal posterior cerebral artery (PCA) in the ICAS-positive group were lower than those in the ICAS-negative group. In contrast, the MMR values of the left pial ACA in the ICAS-positive group were higher.

The mean areas of both ICA and left basal ACA were lower in the ICAS-positive group; however, the mean area of the right vertebral artery (VA) was higher.

Furthermore, the patients who were ICAS-positive exhibited higher mean values of curvature in both ICA, both basal MCA, and left cerebellar artery (Cbll) (Supplementary Table 4).

### Exploring the association between ischaemic stroke and global features

**Figure 2:**
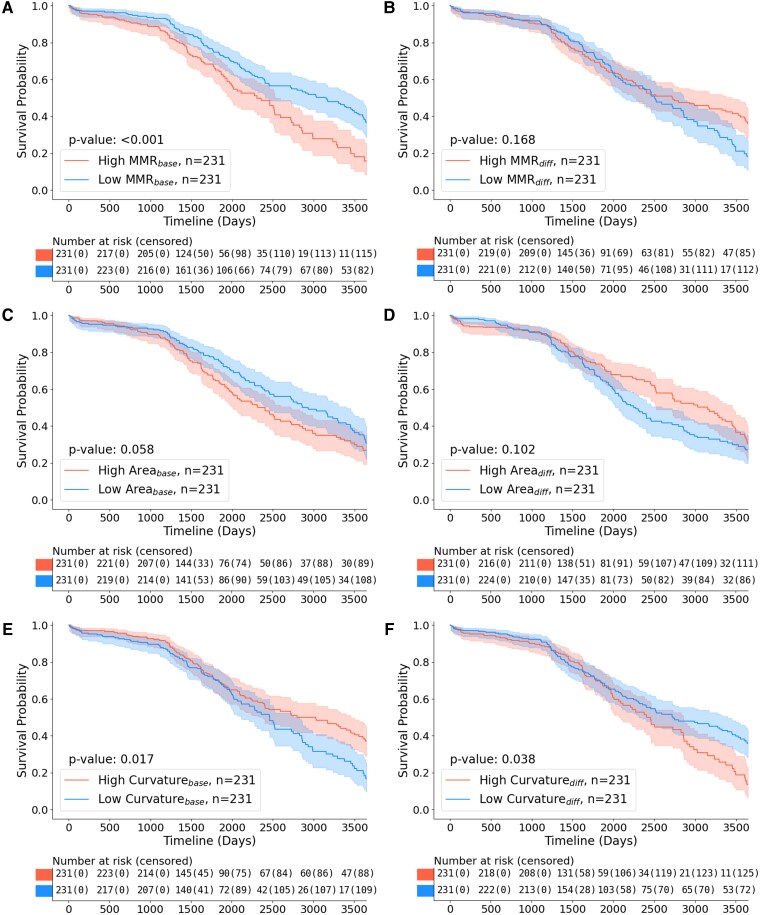
**Kaplan–Meier curves of the 10-year incidence of ischaemic stroke for global features in the 462 patients.** Each plot shows the curves for two groups: ‘High’ and the red patch in the number-at-risk table refers to participants who had values of global features above the median. Between-group differences were assessed using the log-rank test; *P*-values are reported on each panel. ‘Low’ and the blue patch refers to those with values below the median. The observation period was defined as the time interval between the baseline and follow-up time-of-flight (TOF), with the 10-year occurrence of acute ischaemic stroke following the baseline TOF defined as an event. The results are presented for selected metrics, with one metric chosen to represent each of luminal shape, luminal size, and geometric curvature, showing both baseline and annual differences as follows: baseline minimum-maximum diameter ratio (**A**), annual difference in minimum-maximum diameter ratio (**B**), baseline area (**C**), annual difference in area (**D**), baseline curvature (**E**), and annual difference in curvature (**F**). MMR, minimum-maximum diameter ratio; *_base_*, baseline; *_diff_*, annual difference.

The Kaplan–Meier curves of the high- and low-value groups revealed significant differences. MMR*_base_* (*P* < 0.001), LC*_base_* (*P* = 0.003), perimeter*_base_* (*P* = 0.047), MaxD*_base_* (*P* = 0.041) and curvature*_diff_* (*P* = 0.038) were associated with a higher risk of ischaemic stroke in the high-value group. In contrast, curvature*_base_* (*P* = 0.017) and LC*_diff_* (*P* = 0.018) were associated with a higher risk in the low-value group (Fig. 2, Supplementary Figs 3 and 4).

Cox proportional hazards analysis revealed that the 10-year incidence of ischaemic stroke was associated with the following global features: MMR*_base_* [hazard ratio (HR) (95% CI): 1.51 (1.29–1.76); *P* < 0.001], LC*_base_* [HR (95% CI): 1.39 (1.19–1.64); *P* < 0.001], MaxD*_base_* [HR (95% CI): 0.88 (0.77–0.99); *P* = 0.039] and curvature*_base_* [HR (95% CI): 0.04 (0.01–0.19); *P* < 0.001] [Table 3].

**Table 3:** Cox proportional hazards analysis results for the 10-year incidence of ischaemic stroke based on the z-scores of the global features adjusted for sex and age in 462 patients

Global features	HR (95% CI)	*P*-value
Baseline minimum–maximum diameter ratio	1.51 (1.29–1.76)	<0.001
Baseline luminal circularity	1.39 (1.19–1.64)	<0.001
Annual difference of maximum diameter	1.05 (0.90–1.22)	0.556
Annual difference of minimum diameter	1.03 (0.88–1.21)	0.711
Annual difference of perimeter	1.02 (0.87–1.18)	0.845
Annual difference of maximum inscribed sphere radius	1.01 (0.86–1.18)	0.930
Annual difference of area	0.99 (0.85–1.15)	0.921
Baseline maximum inscribed sphere radius	0.96 (0.85–1.08)	0.495
Baseline minimum diameter	0.94 (0.84–1.06)	0.332
Baseline area	0.92 (0.81–1.04)	0.168
Annual difference of minimum-maximum diameter ratio	0.91 (0.76–1.08)	0.279
Baseline perimeter	0.89 (0.79–1.01)	0.064
Annual difference of curvature	0.88 (0.78–1.00)	0.056
Baseline maximum diameter	0.88 (0.77–0.99)	0.039
Annual difference of luminal circularity	0.84 (0.70–1.00)	0.055
Baseline curvature	0.04 (0.01–0.19)	<0.001

The perimeter*_base_* and MaxD*_base_* exhibited conflicting correlations in the Kaplan–Meier and Cox proportional hazards analyses. This discrepancy may have been influenced by age, a known risk factor for ischaemic stroke,^3,4^ which was significantly higher in the high-value group of luminal size metrics (Supplementary Table 5).

### Subgroup analysis of the association between ischaemic stroke and chunk features using ICAS

MMR, area and curvature analyses revealed positive correlations with Bonferroni-adjusted *P* < 0.0031 for MMR_base_ of both pial MCA, left pial ACA, left basal PCA, and left Cbll in the total study population of 462 patients. In contrast, curvature_base_ of the left basal and pial MCA, BA and both basal PCA exhibited significant negative associations.

**Figure 3:**
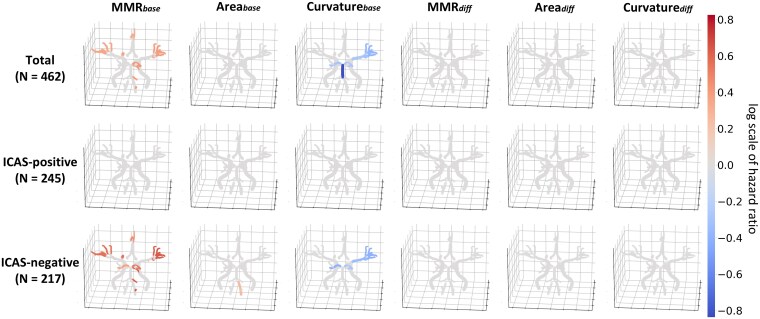
**Vascular mapping of hazard ratios for the 10-year incidence of ischaemic stroke in all patients and subgroups based on intracranial stenosis.** For all patients (*n* = 462), the group with significant stenosis (*n* = 245), and the group without significant stenosis (*n* = 217), chunk features were standardized using z-scores, and hazard ratios were estimated through Cox proportional hazards regression, adjusted for sex and age. The 10-year incidence of acute ischaemic stroke following the baseline time-of-flight (TOF) was defined as the event, with the observation period spanning from baseline to the follow-up TOF. Results with Bonferroni-adjusted *P*-values of <0.0031, as determined by the Wald test, were visualized using colours on the map. The colour bar on the right represented the magnitude of the hazard ratios, with red indicating positive correlations and blue indicating negative correlations. The findings included baseline and annual differences in the minimum-maximum diameter ratio, area, and curvature. MMR, minimum–maximum diameter ratio; LC, luminal circularity; *_base_*, baseline; *_diff_*, annual difference.

The ICAS-positive group (*n* = 245) exhibited no significant correlations. In contrast, most of the correlation patterns observed in the total patients were shared among the patients in the ICAS-negative group (*n* = 217). The MMR_base_ of both pial MCA, left pial ACA, both basal PCA and left Cbll exhibited significant positive correlations. In contrast, the curvature_base_ of the left basal and pial MCA and both basal PCA exhibited significant negative correlations (Figure 3).

Tables S6–S8 present the exact values of HR, 95% CI, and *P*-values for each variable.

## Discussion

The present study identified the morphological features of cerebral arteries associated with ischaemic stroke. The global features, MMR_base_ and LC_base,_ exhibited positive correlations with ischaemic stroke, whereas curvature_base_ demonstrated a negative correlation. Chunk-based feature analysis, which accounted for vascular distribution, revealed that MMR_base_ exhibited a positive correlation with small arteries, whereas curvature_base_ exhibited negative correlations in regions of the posterior circulation and left MCA. Although similar correlation patterns were observed in the ICAS-negative group, no significant correlations were observed in the ICAS-positive group.

### Atherosclerotic features and plaque distribution in cerebral arteries

Complex and heterogeneous factors influence the morphological features of the cerebral arteries. Specifically, atherosclerosis which is the primary cause of luminal narrowing in large arteries must be taken into consideration during the evaluation of the independent correlation of each feature with ischaemic stroke.^12-14^ Although statistical significance varied across the results, lower MMR_base_; smaller area_base_ and higher curvature_base_ in large arteries, including the ICA, basal MCA, VA and BA, were observed in the ICAS-positive group, which is characterized by >50% of stenosis,^8^ suggesting a potential relationship between these features and atherosclerosis (Supplementary Table 4).

Atherosclerosis can alter the luminal shape, leading to deviations in circularity. A previous study examining the association between intracranial arterial wall thickening patterns and underlying pathology reported that atherosclerotic plaques were associated with eccentric wall thickening and that concentric wall thickening was predominantly linked to inflammatory processes.^15^ Furthermore, atherosclerotic burden exhibited an independent association with tortuosity, defined as the degree of twisting or bending of a blood vessel and quantified as the curvature in this study.^16^ This association can be attributed to tortuosity inducing changes in velocity and turbulence in blood flow. Altered blood flow in tortuous vessels can create regions of low oscillatory shear stress that are closely associated with endothelial dysfunction. These changes increase the susceptibility of these areas to the formation of atherosclerotic plaques.^16-18^

The variability in plaque distribution based on vascular geometry indicates greater susceptibility to atherosclerotic changes in certain arterial segments, emphasizing the importance of the careful analysis of arterial features in specific regions. A previous autopsy study revealed that early and advanced plaques were more frequently observed in large arteries such as the ICA, BA, VA and MCA.^19^ Another study that identified stenosis of >50% in the general population using TOF imaging reported the highest frequency in the ICA, followed by the VA, PCA and MCA.^20^ The highest frequency of ICAS was observed in the basal MCA and ICA in the present study, followed by the basal PCA and basal ACA (Supplementary Table 4). However, plaques without luminal stenosis are difficult to detect on TOF imaging. Furthermore, the differences between the study populations likely explain the discrepancies observed in previous studies.

### Arterial stiffness and age-related metrics

Arterial stiffness, a key index of vascular aging, is associated with cardiovascular disease (CVD) risk and varies in terms of its impact on arterial morphology depending on vascular geometry.^21^ Stiffness accelerates with age and elevated blood pressure in large, elastin-rich arteries, referred to as central arteries. Arterial stiffness progresses owing to the damage or degradation of elastic fibres, accumulation of collagen fibres and increased cross-linking of collagen fibres, often accompanied by an increased luminal radius.^22,23^ A healthy central artery expands during systole to store elastic energy in the arterial wall and contracts during diastole to maintain continuous blood flow. This phenomenon is known as the ‘Windkessel effect’.^24^ However, the increased pulsatile pressure induces microvascular damage and hypertrophic remodelling of small arteries as the arterial stiffness increases, resulting in an increase in peripheral resistance. This results in an increase in blood pressure, potentially creating a feedback loop that accelerates stiffness in the central arteries.^23,25^

The present study aimed to identify features that could serve as potential indicators of vascular aging and arterial stiffness by examining the relationship between age and global features. Circular luminal shape and increased luminal size exhibited associations with age (Table 2 and Supplementary Table 5). Arterial stiffness can impair vessel reactivity,^25^ which may inhibit transient changes in the vessel diameter and lead to a rigid circular lumen. The association between increased luminal size and age may be related to the weakening of the arterial wall owing to damage to the elastin fibers during aging.^22,23^ These findings suggest a link between vascular aging and the morphological changes in brain vessels. Thus, these features could serve as important markers for assessing cerebrovascular health.

### Correlation interpretation between arterial features and ischaemic stroke

**Figure 4:**
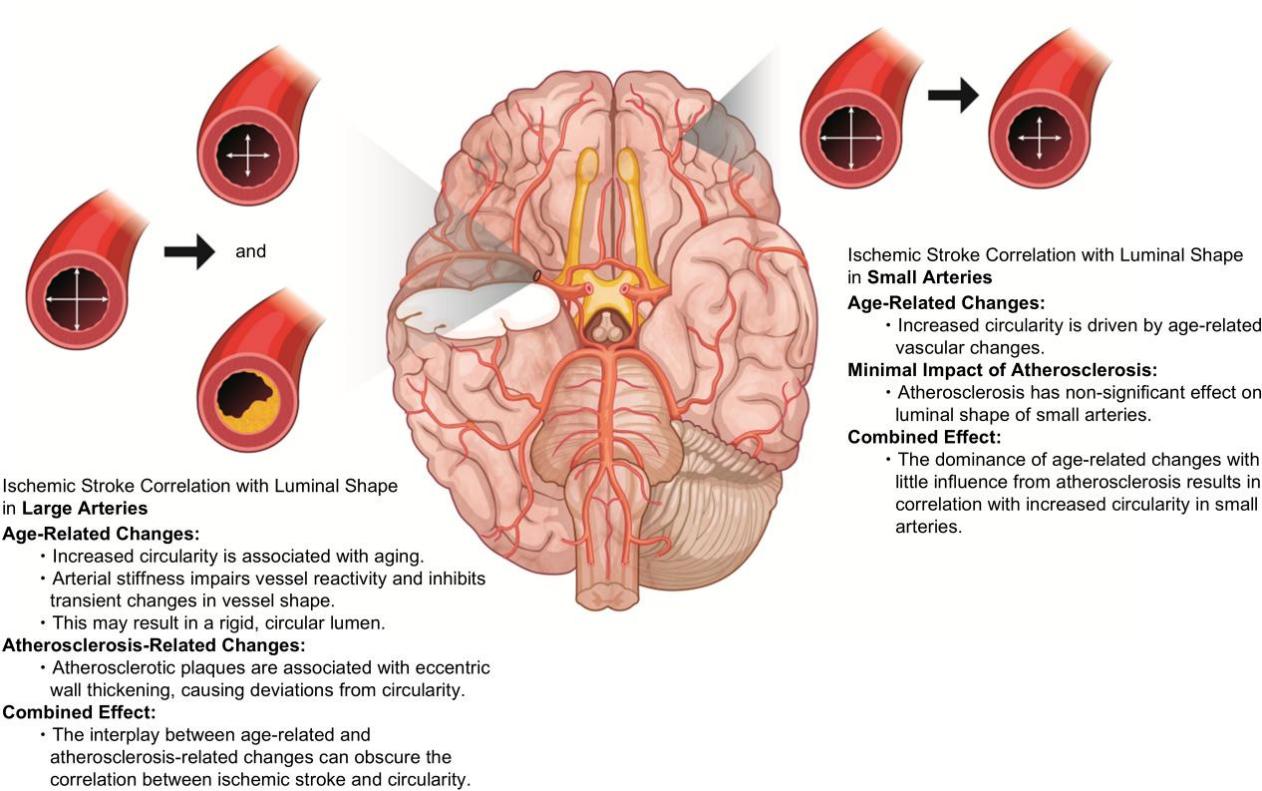
**Correlation of ischaemic stroke with luminal shape in large and small arteries.** Among cerebrovascular morphological features, baseline luminal shape exhibited different correlations with acute ischaemic stroke in large versus small arteries. Increases in luminal circularity—an age-related change that may reflect arterial stiffness—were opposite in direction to the effect of atherosclerosis, which tends to reduce luminal circularity. Atherosclerosis-related luminal alterations were observed predominantly in large arteries; consequently, the correlation between age-related changes and acute ischaemic stroke was attenuated in large arteries but remained pronounced in small arteries.

The positive correlation between the baseline luminal shape metrics and ischaemic stroke observed in small arteries in the present study may be attributed to the increased peripheral resistance and blood pressure caused by arterial stiffness, which further accelerates central artery stiffness through feedback mechanisms.^23^ Small artery remodelling may impair vascular reactivity and reduce reactive hyperaemia in response to ischaemic stimuli, thereby heightening stroke susceptibility.^25^ Furthermore, small arteries may be relatively resistant to atherosclerosis based on plaque distribution.^19^ The findings of the present study indicate that ICAS reduces luminal shape metrics in large arteries, potentially obscuring the positive correlation between ischaemic stroke and luminal shape metrics. However, the morphological changes associated with the ICAS burden were not significant in the small arteries, emphasizing a stronger correlation with age-related vascular changes in these vessels (Fig. 4).

The baseline luminal size metrics exhibited no significant associations with ischaemic stroke in most chunks in the chunk analysis. A relationship between severe stenosis and recurrent ischaemic stroke was observed in previous studies^7^; however, increased plaque vulnerability in patients with positive remodelling highlights the limitations of using luminal size metrics alone to predict ischaemic stroke.^26,27^ Furthermore, the opposing directional trends of the size metrics associated with the ICAS-positive group compared with those associated with age-related changes may have influenced the results.

The negative correlation between baseline curvature and ischaemic stroke presents an interpretative challenge. Previous studies that proposed models to predict cerebrovascular age based on morphological features of the brain vessels have reported a positive correlation between curvature and age.^28^ Moreover, other studies have reported an association between tortuosity and atherosclerosis.^16^ However, a study on the role of aortic geometry in stroke propensity reported that atrial fibrillation-induced stroke propensity increases significantly in patients with lower aortic curvature, whereas the propensity for alteration is negligible in those with higher curvature.^29^ These findings indicate that the impact of curvature on stroke varies depending on specific conditions, highlighting the requirement for a deeper understanding of the haemodynamic effects of low curvature in stroke mechanisms.

In the present study, significant associations between luminal shape metrics and ischaemic stroke were observed in the ICAS-negative group but not in the ICAS-positive group. Several factors may account for this discrepancy. First, luminal shape metrics tended to increase with age but decrease in association with atherosclerotic changes. This pattern suggests that the relationship between luminal morphology and stroke risk may have been attenuated in the ICAS-positive group, where advanced atherosclerosis is more prevalent. In addition, a deviation of luminal shape from circularity does not necessarily indicate an increased plaque burden. Previous studies have demonstrated that focal plaques involving less than 50% of the vessel wall often exhibit an eccentric thickening pattern, whereas concentric plaques typically demonstrate a more diffuse growth pattern.^30^ Such structural variations may have contributed to the attenuated associations in ICAS-positive group.

The absence of a significant correlation between the annual differences in arterial features and ischaemic stroke may be attributed to the treatment-induced changes in arterial features among patients with a history of ischaemic stroke, TIA or stroke risk factors, which could complicate this relationship. For instance, endovascular interventions can lead to arterial remodelling, including expansion of the inner diameter and changes in the arterial wall.^31^ Experimental animal studies have shown that clopidogrel inhibits endothelial dysfunction and vascular remodelling.^32^ Statins also improve arterial elasticity and stabilize atherosclerotic plaques in the coronary arteries,^33^ reduce stenosis severity and decrease plaque enhancement volume in patients with symptomatic intracranial atherosclerotic disease.^34^ Furthermore, antihypertensive medications may also indirectly contribute by lowering the mean arterial pressure, thereby reducing arterial stiffness. Notably, angiotensin-converting enzyme (ACE) inhibitors and angiotensin receptor blockers (ARBs) reduce the carotid-femoral pulse wave velocity (cf-PWV) to a greater extent than other antihypertensive agents with similar blood pressure-lowering effects.^23,35^ These diverse therapeutic effects likely exert a combined effect on the changes in arterial features, potentially masking or attenuating the correlation between ischaemic stroke and annual differences in arterial features.

### Strengths and limitations

The present study conducted a chunk feature analysis of the entire cerebral artery while accounting for vascular distribution to explore the correlation between arterial features and ischaemic stroke. The potential predictive markers associated with ischaemic stroke were systematically explored by performing a comprehensive analysis that encompasses the entire brain rather than being limited to specific arteries. The use of the in-house software program, which facilitated automated cerebral artery labelling, enabled high-throughput analysis. This allowed for the quantification of various arterial metrics across different chunks for each patient, achieving greater consistency and reproducibility through algorithm-based analysis.^5^ Subgroup analyses were conducted to account for the relationship between ICAS burden and ischaemic stroke, after adjusting for age and sex to evaluate the independent association between arterial features and ischaemic stroke more accurately.^12,36,37^

Nevertheless, this study has some limitations warranting further consideration. TOF images are reconstructed based on blood flow, which can result in distortions in the vessels affected by stenosis or complex blood flow patterns.^38^ In addition, TOF imaging provides limited information regarding wall thickness and plaque vulnerability, making the accurate evaluation of arterial remodelling or plaque characteristics challenging. However, given the absence of tools that can perfectly replicate the actual vascular structure and its noninvasive nature, TOF remains a suitable imaging modality for analysis. Future studies must incorporate methods that provide supplementary information regarding wall thickness and plaque characteristics to address these limitations.

Another limitation is the heterogeneity in baseline definition, as some patients had TOF imaging at registry enrollment while others had earlier scans. Subgroup analyses stratified by baseline status showed broadly similar trends (Supplementary Fig. 5, Supplementary Tables 9 and 10), suggesting minimal impact on the overall findings; however, this variability should be considered when interpreting the results.

The single-centre nature, relatively small sample size, and the potential for selection bias limited the findings of the study. The study population may not be representative of the general population, and caution must be exercised while generalizing these findings.

As the study enrolled patients with acute ischaemic stroke or TIA within a defined period—a cohort that may differ from the general population—survival bias and baseline heterogeneity may have influenced the observed associations. To address these limitations and enhance generalizability, future research should adopt a prospective, multicentre design. In addition, defining threshold values for key vascular morphological features could sharpen risk stratification and facilitate translation into clinical decision-making.

Selection bias related to follow-up MRA referral should also be considered. Decisions to obtain follow-up MRA are likely influenced by clinical factors—such as the severity of intracranial atherosclerotic stenosis, vascular risk factors (e.g. hypertension, diabetes, dyslipidemia), and the patient’s overall clinical status—and may be particularly consequential for analyses of dynamic changes. Accordingly, the interpretation and clinical application of our findings should be situated within the clinical context in which follow-up MRA is ordered and performed. In future work, we will prospectively ascertain and standardize the indications and clinical context for follow-up MRA and incorporate these factors into study design and analytic models to minimize referral-related selection bias.

## Conclusion

A comprehensive quantitative analysis of the morphological characteristics of individual cerebrovascular arteries was performed using an in-house vessel analysis software program in the present study. Examination of the entire cerebrovascular system revealed significant correlations between the arterial features and ischaemic stroke. Furthermore, the stratification of patients based on the presence of significant stenosis enabled adjustment for ICAS, thereby facilitating the assessment of independent associations between the arterial features and ischaemic stroke. Notably, the associations between specific cerebrovascular morphological features and acute ischaemic stroke were preserved in the ICAS-negative subgroup, suggesting that these imaging-derived metrics may be particularly informative for refining secondary prevention among individuals who might otherwise appear at lower risk.

This methodology provides a systematic and objective framework for evaluating cerebral arterial features and emphasizes the clinical potential of integrating imaging metrics, clinical parameters, and vascular distribution in ischaemic stroke research. The ultimate goal of this study is to develop a stroke risk assessment model that incorporates vascular morphological features, thereby enabling earlier identification and intervention for high-risk individuals. The findings of the present study support the advancement of personalized risk assessment and therapeutic strategies, thereby enabling a more precise and effective approach to cerebrovascular care.

## Supplementary Material

fcag037_Supplementary_Data

## Data Availability

The data supporting this study are not publicly available because of privacy concerns specific to our centre. Publicly sharing data can compromise the confidentiality of patient information. All analyses were performed in Python; the corresponding code is provided in the Supplementary material (Supplementary_material_python_code). For further inquiries, the corresponding author may be contacted.
