# Supplementary material for: Association of cerebrovascular morphology with ischaemic stroke considering intracranial stenosis
Source: Brain Commun. 2026 Feb 10;8(2):fcag037. doi: 10.1093/braincomms/fcag037 (PMC13044395; doi:10.1093/braincomms/fcag037)
Supplement: fcag037_Supplementary_Data [file fcag037_supplementary_data.zip › Supplementary_materials.pdf]

Supplementary Material

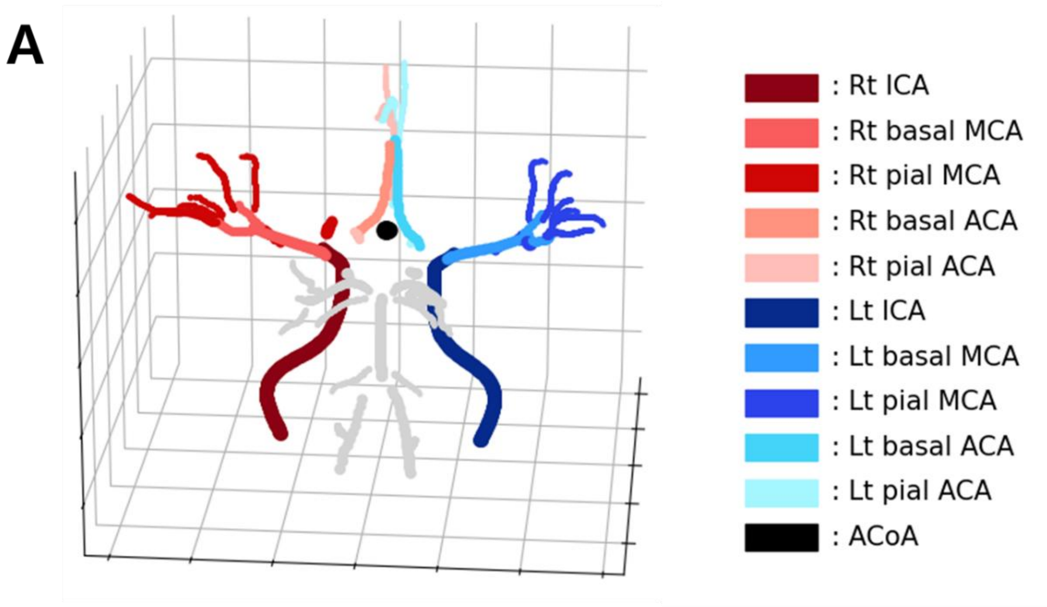

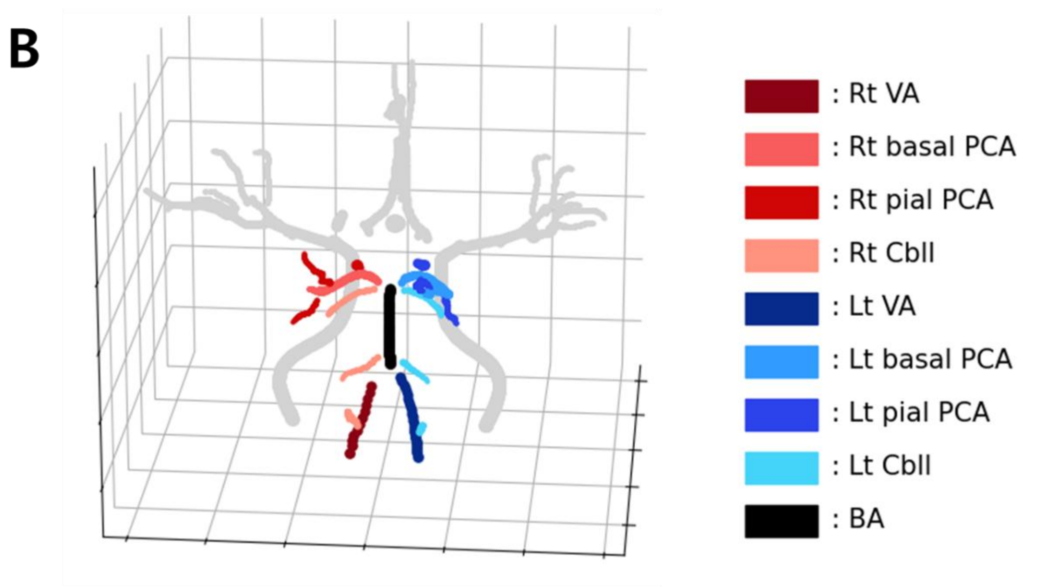

Supplementary Figure 1. Vascular mapping of the chunks.
(A) Anterior circulation chunks, (B) Posterior circulation chunks
Rt, Right; Lt, Left; ICA, internal carotid artery; MCA, middle cerebral artery; ACA, anterior cerebral artery; ACoA, anterior communicating artery; VA, vertebral artery; PCA, posterior cerebral artery; Cbll, cerebellar artery; BA, basilar artery.

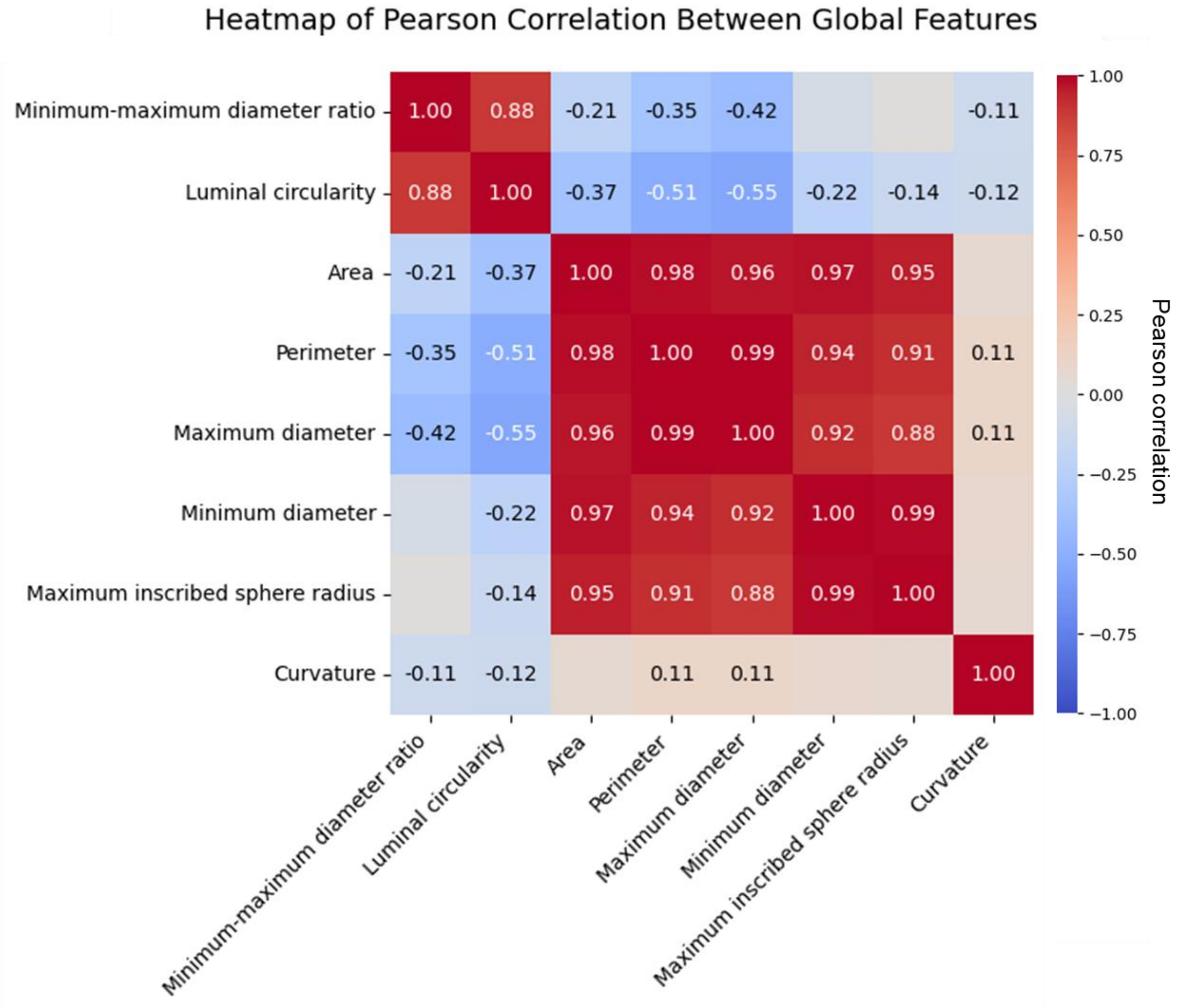

Supplementary Figure 2. Heatmap of correlation between the global features in the 462 patients. Pearson's correlation analysis was performed to illustrate the correlations between the baseline global features, with the correlation strength represented by the color scale on the accompanying color bar. P-values were obtained from two-sided Student's t-tests. Only correlation values for significant results with a p-value of <0.05 are presented in the cells.

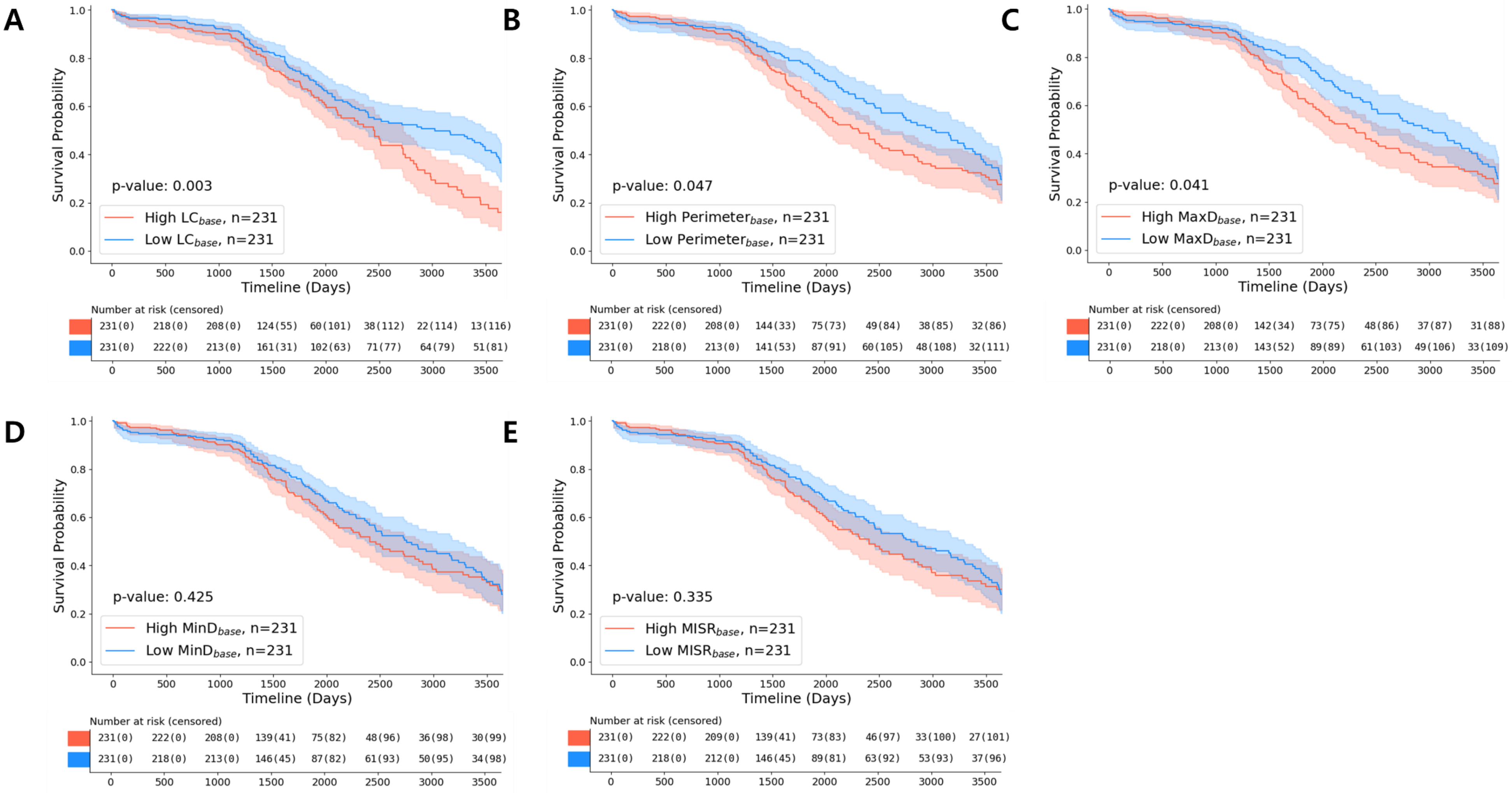

Supplementary Figure 3. Kaplan–Meier curves of the 10-year incidence of ischemic stroke for baseline global features in the 462 patients. Each plot showed curves for two groups: 'High' (red patch in the number-at-risk table) and 'Low' (blue patch in the number-at-risk table) as described in Figure 2. Between-group differences were assessed using the log-rank test; p-values are reported on each panel. The results are presented for the following baseline metrics: luminal circularity (A), perimeter (B), maximum diameter (C), minimum diameter (D), and maximum inscribed sphere radius (E).

LC, luminal circularity; MaxD, maximum diameter; MinD, minimum diameter; MISR, maximum inscribed sphere radius; $_{base}$, baseline; $_{diff}$; annual difference

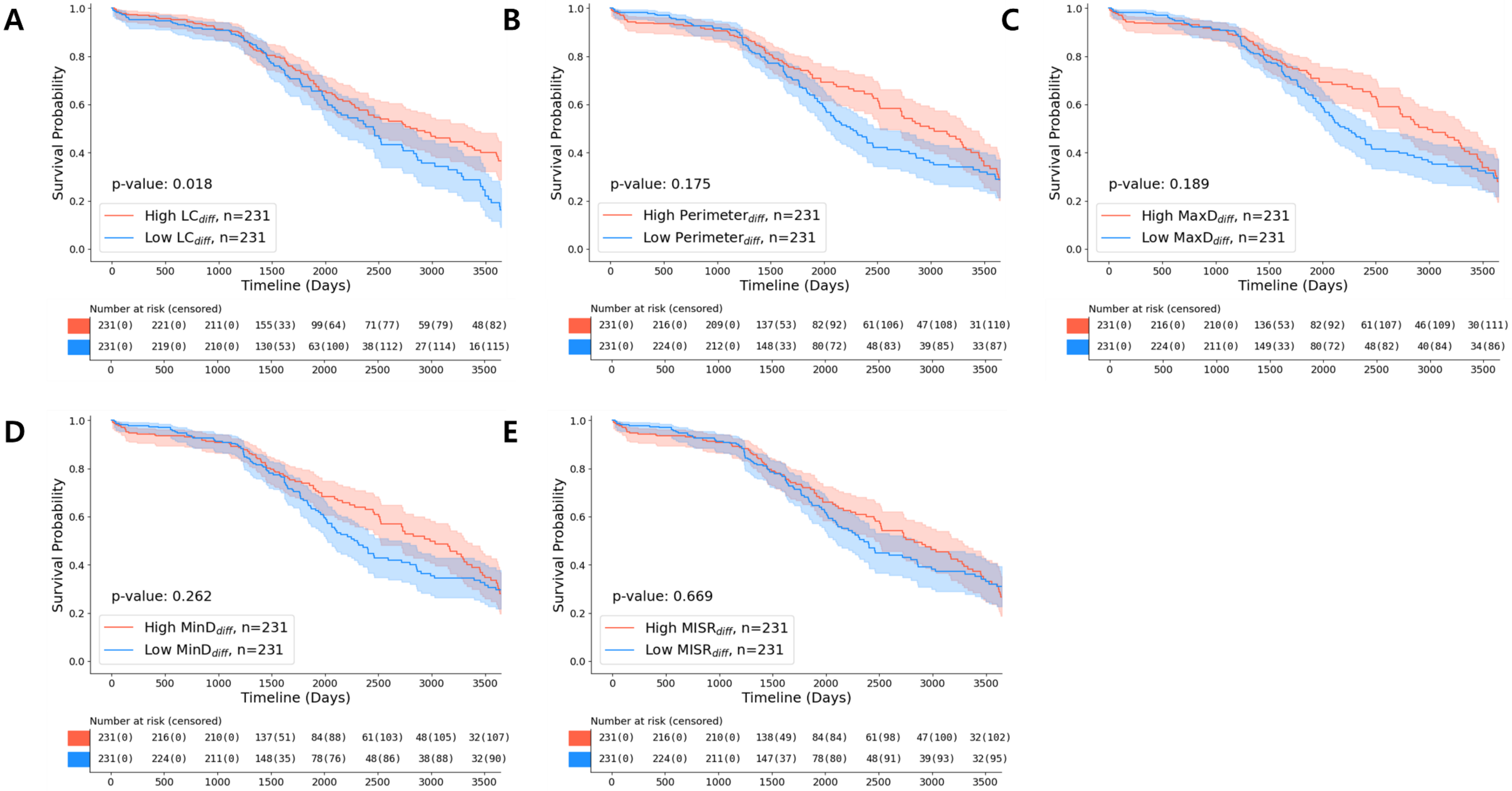

Supplementary Figure 4. Kaplan–Meier curves of the 10-year incidence of ischemic stroke for annual difference of global features in the 462 patients. Each plot showed curves for two groups: 'High' (red patch in the number-at-risk table) and 'Low' (blue patch in the number-at-risk table) as described in Figure 2. Between-group differences were assessed using the log-rank test; p-values are reported on each panel. Results are presented for the following additional metrics of annual differences: luminal circularity (A), perimeter (B), maximum diameter (C), minimum diameter (D), and maximum inscribed sphere radius (E).

LC, luminal circularity; MaxD, maximum diameter; MinD, minimum diameter; MISR, maximum inscribed sphere radius; $_{base}$, baseline; $_{diff}$, annual difference.

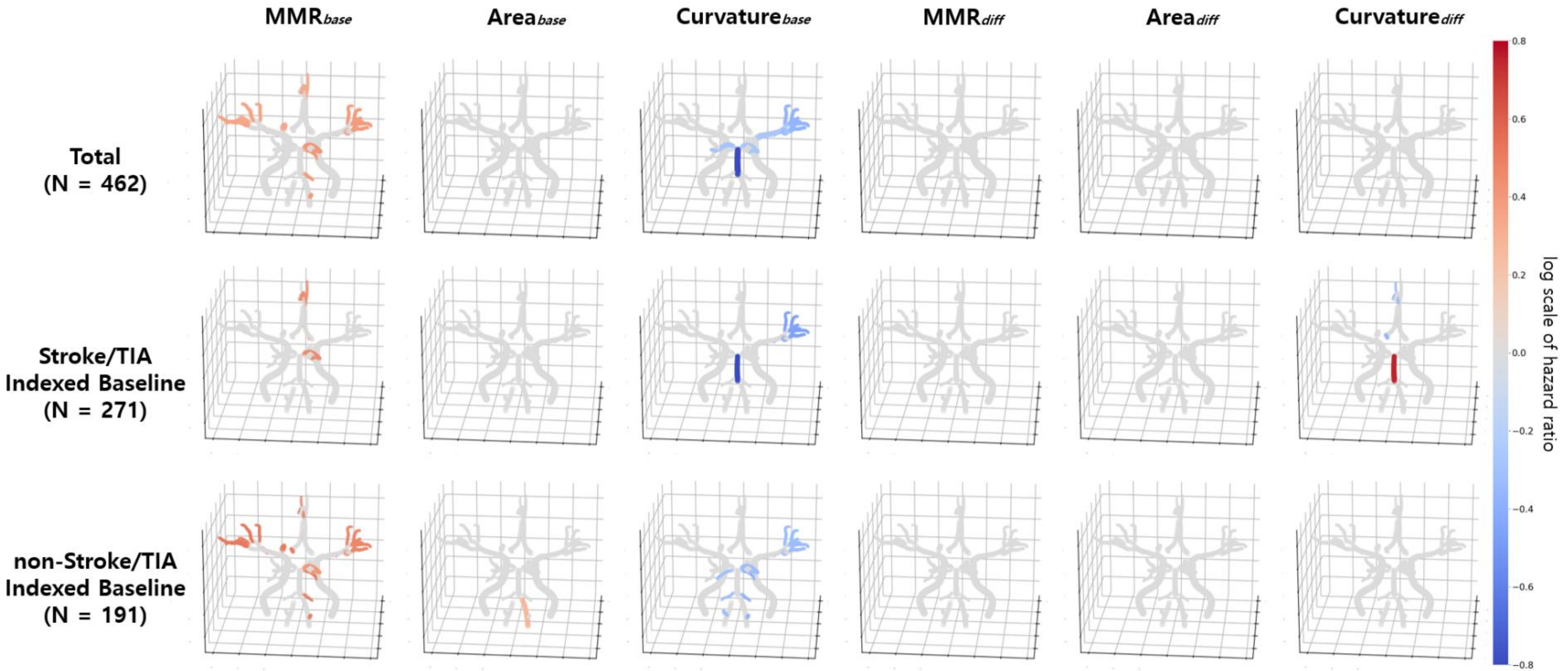

Supplementary Figure 5. Vascular mapping of hazard ratios for the 10-year incidence of ischemic stroke in all patients and subgroups stratified by baseline status. Analyses included all patients (n = 462), those with a stroke/transient ischemic stroke (TIA)-indexed baseline (n = 271), and those with a non-indexed baseline (n = 191). Chunk features were standardized using z-scores, and hazard ratios were estimated with Cox proportional hazards models adjusted for age and sex. The event was defined as the occurrence of an acute ischemic stroke within 10 years after the baseline time-of-flight (TOF) scan, and the observation period extended from baseline to the subsequent TOF imaging. Associations that met the Bonferroni-corrected significance threshold ($p < 0.0031$, Wald test) were shown on the map, with the color bar indicating the magnitude of hazard ratios: red for positive and blue for negative associations. Key findings included both baseline values and annual changes in the minimum-maximum diameter ratio, area, and curvature.

TIA, transient ischemic attack; MMR, minimum–maximum diameter ratio; $_{base}$, baseline; $_{diff}$, annual difference

Supplementary Table 1. Acquisition Parameters of time-of flight at Baseline and Follow-up

Manufacturer	Baseline (n=462)	Follow-up (n=462)
Philips Medical Systems / Philips Healthcare	419 (90.7)	420 (90.9)
Siemens Healthineers	17 (3.7)	42 (9.1)
GE Medical Systems	26 (5.6)	–
**Echo time (ms)**	**Baseline**	**Follow-up**
3.45	414 (89.61)	418 (90.48)
6.90–6.91	26 (5.63)	1 (0.22)
3.34	10 (2.16)	20 (4.33)
3.69	5 (1.08)	21 (4.55)
3.42	2 (0.43)	1 (0.22)
Others	5 (1.08)	1 (0.22)
**Repetition time (ms)**	**Baseline**	**Follow-up**
25	397 (85.93)	372 (80.52)
33–34	25 (5.41)	–
21	13 (2.81)	21 (4.55)
24	11 (2.38)	–
23	10 (2.16)	32 (6.93)
22	5 (1.08)	21 (4.55)
Others	1 (0.22)	16 (3.46)
**Flip Angle (°)**	**Baseline**	**Follow-up**
20	435 (94.16)	417 (90.26)
18	26 (5.63)	24 (5.19)
Others	1 (0.22)	21 (4.55)
**Field of view (mm)**	**Baseline**	**Follow-up**
250 × 250	395 (85.50)	404 (87.45)
200 × 200	23 (4.98)	3 (0.65)
220 × 220	19 (4.11)	13 (2.81)
195 × 220	10 (2.16)	39 (8.87)
Others	15 (3.25)	3 (0.65)
**Voxel matrix (mm^3)**	**Baseline**	**Follow-up**
880 × 880 × 380	201 (43.51)	370 (80.09)
880 × 880 × 338	129 (27.92)	8 (1.73)
1024 × 1024 × (3–5)	51 (11.04)	4 (0.87)
704 × 704 × 338	21 (4.55)	3 (0.65)
Others	60 (12.99)	77 (16.66)
**Acquisition time (s)**	**Baseline**	**Follow-up**
352	155 (33.55)	143 (30.95)
310–311	94 (20.35)	8 (1.73)
347–349	25 (5.41)	76 (16.45)
230–231	1 (0.22)	91 (19.70)
Others	69 (14.93)	100 (21.65)
NaN	118 (25.54)	44 (9.52)
**Pixel Bandwidth**	**Baseline**	**Follow-up**
124	366 (79.22)	366 (79.22)
122–123	32 (6.93)	3 (0.65)
220	10 (2.16)	20 (4.33)
108–109	10 (2.16)	2 (0.43)
Others	23 (4.98)	71 (15.37)
NaN	21 (4.55)	–

Supplementary Table 2. Evaluation of arterial features using in-house vessel analysis software.[1]

Arterial features	Evaluation
Minimum-maximum diameter ratio	Ratio of the maximum to the minimum diameter within an arterial segment.
Luminal circularity	A measure of arterial lumen's roundness, calculated as $(4\pi \times \text{area}) / \text{perimeter}^2$
Area	Cross-sectional area of an arterial segment.
Perimeter	The length of the continuous boundary line around an arterial cross-section.
Maximum diameter	The largest diameter within a defined arterial segment.
Minimum diameter	The smallest diameter within a defined arterial segment.
Maximum inscribed sphere radius	The radius of the largest sphere that fits entirely within the arterial segment and is tangent to the luminal surface.
Curvature	The degree of deviation from a straight, cylindrical arterial course.

Supplementary Table 3. Comparison between the baseline global features of the ICAS-positive (n = 245) and ICAS-negative (n = 217) groups.

Global features	ICAS-positive	ICAS-negative	p-value
Minimum-maximum diameter ratio	0.827 ± 0.024	0.830 ± 0.027	0.170
Luminal circularity	0.963 ± 0.007	0.963 ± 0.009	0.703
Area	7.559 ± 1.578	7.802 ± 1.495	0.091
Perimeter	9.270 ± 0.993	9.350 ± 0.970	0.379
Maximum diameter	3.150 ± 0.347	3.170 ± 0.340	0.531
Minimum diameter	2.596 ± 0.260	2.625 ± 0.237	0.205
Maximum inscribed sphere radius	1.287 ± 0.119	1.302 ± 0.111	0.143
Curvature	0.312 ± 0.375	0.282 ± 0.028	0.243

ICAS, intracranial atherosclerotic stenosis

Supplementary Table 4. Comparison between the baseline chunk features of the ICAS-positive (n = 245) and ICAS-negative (n = 217) groups

Chunks	Counts of significant stenosis	Minimum-maximum diameter ratio			Area			Curvature		
		ICAS-positive	ICAS-negative	p-value	ICAS-positive	ICAS-negative	p-value	ICAS-positive	ICAS-negative	p-value
Rt ICA	79	0.883 ± 0.035	0.894 ± 0.044	0.004	19.690 ± 5.209	22.418 ± 4.422	<0.001	0.256 ± 0.087	0.240 ± 0.035	0.008
Lt ICA	88	0.882 ± 0.053	0.896 ± 0.022	<0.001	20.113 ± 5.850	22.431 ± 4.321	<0.001	0.257 ± 0.102	0.239 ± 0.028	0.016
Rt basal MCA	99	0.829 ± 0.066	0.844 ± 0.069	0.021	9.056 ± 2.433	9.363 ± 2.667	0.214	0.281 ± 0.059	0.263 ± 0.045	<0.001
Lt basal MCA	76	0.839 ± 0.067	0.856 ± 0.060	0.008	9.002 ± 3.830	9.425 ± 2.688	0.188	0.275 ± 0.055	0.257 ± 0.055	0.001
Rt basal ACA	36	0.744 ± 0.144	0.743 ± 0.143	0.942	9.467 ± 4.775	10.362 ± 6.801	0.134	0.306 ± 0.116	0.298 ± 0.115	0.496
Lt basal ACA	35	0.770 ± 0.102	0.753 ± 0.129	0.139	8.443 ± 2.678	9.631 ± 3.551	<0.001	0.288 ± 0.060	0.300 ± 0.149	0.270
Rt pial MCA	–	0.844 ± 0.038	0.846 ± 0.038	0.496	3.683 ± 1.234	3.671 ± 1.263	0.915	0.293 ± 0.038	0.315 ± 0.342	0.318
Lt pial MCA	–	0.845 ± 0.046	0.850 ± 0.036	0.252	3.729 ± 1.810	3.580 ± 0.977	0.283	0.290 ± 0.044	0.286 ± 0.036	0.267
Rt pial ACA	–	0.804 ± 0.089	0.810 ± 0.084	0.475	4.548 ± 2.497	4.620 ± 2.386	0.768	0.303 ± 0.069	0.297 ± 0.092	0.449
Lt pial ACA	–	0.828 ± 0.059	0.812 ± 0.082	0.019	3.954 ± 1.470	4.350 ± 3.846	0.150	0.295 ± 0.057	0.297 ± 0.073	0.727
Rt VA	27	0.849 ± 0.124	0.864 ± 0.103	0.255	13.226 ± 6.444	11.870 ± 4.630	0.042	0.422 ± 0.857	0.310 ± 0.307	0.133
Lt VA	36	0.874 ± 0.090	0.882 ± 0.099	0.434	13.155 ± 5.793	13.590 ± 6.215	0.502	0.288 ± 0.261	0.281 ± 0.323	0.832
BA	15	0.880 ± 0.105	0.912 ± 0.047	<0.001	14.007 ± 8.200	14.114 ± 4.210	0.873	0.302 ± 0.992	0.202 ± 0.056	0.164
Rt Cbll	–	0.814 ± 0.072	0.820 ± 0.070	0.385	3.822 ± 1.712	3.958 ± 2.632	0.511	0.329 ± 0.070	0.346 ± 0.434	0.560
Lt Cbll	–	0.799 ± 0.089	0.801 ± 0.097	0.856	4.238 ± 3.702	4.360 ± 5.051	0.773	0.346 ± 0.121	0.317 ± 0.085	0.004
Rt basal PCA	44	0.831 ± 0.051	0.843 ± 0.038	0.006	6.062 ± 2.822	5.693 ± 1.576	0.095	0.295 ± 0.048	0.286 ± 0.050	0.061
Lt basal PCA	42	0.836 ± 0.044	0.840 ± 0.042	0.381	5.714 ± 2.259	5.568 ± 1.417	0.420	0.287 ± 0.036	0.283 ± 0.042	0.290
Rt pial PCA	–	0.812 ± 0.052	0.814 ± 0.074	0.793	3.510 ± 1.395	3.416 ± 1.554	0.584	0.335 ± 0.089	0.340 ± 0.100	0.645
Lt pial PCA	–	0.800 ± 0.111	0.815 ± 0.082	0.206	3.602 ± 2.824	3.452 ± 2.095	0.627	0.355 ± 0.211	0.333 ± 0.080	0.273

ICAS, intracranial atherosclerotic stenosis; Rt, Right; Lt, Left; ICA, internal carotid artery; MCA, middle cerebral artery; ICA, internal carotid artery; ACA, anterior cerebral artery; VA, vertebral artery; BA, basilar artery; Cbll, cerebellar artery; PCA, posterior cerebral artery.

Supplementary Table 5. Clinical profiles stratified into the high and low groups of baseline global features.

Global features	Age			Sex (male)			Body mass index		
	High group	Low group	p-value	High group	Low group	p-value	High group	Low group	p-value
Minimum-maximum diameter ratio	66.07 ± 13.18	62.64 ± 13.07	0.005	156 (67.53)	141 (61.04)	0.174	24.89 ± 3.75	24.30 ± 3.74	0.091
Luminal circularity	65.83 ± 13.41	62.88 ± 12.88	0.016	149 (64.50)	148 (64.07)	1.000	24.93 ± 3.63	24.26 ± 3.85	0.056
Area	67.51 ± 12.09	61.20 ± 13.58	<0.001	160 (69.26)	137 (59.31)	0.033	24.51 ± 3.90	24.67 ± 3.61	0.655
Perimeter	67.18 ± 11.88	61.53 ± 13.90	<0.001	156 (67.53)	141 (61.04)	0.174	24.56 ± 3.87	24.62 ± 3.65	0.867
Maximum diameter	67.04 ± 12.09	61.67 ± 13.77	<0.001	155 (67.10)	142 (61.47)	0.244	24.63 ± 3.98	24.55 ± 3.53	0.831
Minimum diameter	67.35 ± 12.07	61.36 ± 13.66	<0.001	158 (68.40)	139 (60.17)	0.081	24.58 ± 3.96	24.60 ± 3.55	0.964
Maximum inscribed sphere radius	67.26 ± 12.01	61.45 ± 13.75	<0.001	158 (68.40)	139 (60.17)	0.081	24.51 ± 3.98	24.67 ± 3.53	0.632
Curvature	63.71 ± 13.06	65.00 ± 13.37	0.295	136 (58.87)	161 (69.70)	0.020	24.55 ± 4.19	24.63 ± 3.27	0.817

Global features	Hypertension			Diabetes mellitus			Dyslipidemia		
	High group	Low group	p-value	High group	Low group	p-value	High group	Low group	p-value
Minimum-maximum diameter ratio	156 (67.53)	159 (68.83)	0.842	70 (30.30)	66 (28.57)	0.759	112 (48.48)	92 (39.83)	0.075
Luminal circularity	159 (68.83)	156 (67.53)	0.842	70 (30.30)	66 (28.57)	0.759	111 (48.05)	93 (40.26)	0.111
Area	166 (71.86)	149 (64.50)	0.110	64 (27.71)	72 (31.17)	0.475	98 (42.42)	106 (45.89)	0.512
Perimeter	167 (72.29)	148 (64.07)	0.072	67 (29.00)	69 (29.87)	0.919	94 (40.69)	110 (47.62)	0.160
Maximum diameter	168 (72.73)	147 (63.64)	0.046	68 (29.44)	68 (29.44)	1.000	99 (42.86)	105 (45.45)	0.640
Minimum diameter	166 (71.86)	149 (64.50)	0.110	61 (26.41)	75 (32.47)	0.185	98 (42.42)	106 (45.89)	0.512
Maximum inscribed sphere radius	166 (71.86)	149 (64.50)	0.110	63 (27.27)	73 (31.60)	0.358	98 (42.42)	106 (45.89)	0.512
Curvature	162 (70.13)	153 (66.23)	0.424	66 (28.57)	70 (30.30)	0.759	102 (44.16)	102 (44.16)	1.000

Global features	Current smoking			Coronary artery disease			Atrial fibrillation		
	High group	Low group	p-value	High group	Low group	p-value	High group	Low group	p-value
Minimum-maximum diameter ratio	42 (18.18)	51 (22.08)	0.353	31 (13.42)	37 (16.02)	0.512	27 (11.69)	29 (12.55)	0.887
Luminal circularity	43 (18.61)	50 (21.65)	0.486	35 (15.15)	33 (14.29)	0.896	26 (11.26)	30 (12.99)	0.669
Area	48 (20.78)	45 (19.48)	0.817	38 (16.45)	30 (12.99)	0.358	35 (15.15)	21 (9.09)	0.064
Perimeter	48 (20.78)	45 (19.48)	0.817	34 (14.72)	34 (14.72)	1.000	35 (15.15)	21 (9.09)	0.064
Maximum diameter	51 (22.08)	42 (18.18)	0.353	35 (15.15)	33 (14.29)	0.896	33 (14.29)	23 (9.96)	0.200
Minimum diameter	49 (21.21)	44 (19.05)	0.643	37 (16.02)	31 (13.42)	0.512	34 (14.72)	22 (9.52)	0.117
Maximum inscribed sphere radius	49 (21.21)	44 (19.05)	0.643	36 (15.58)	32 (13.85)	0.694	33 (14.29)	23 (9.96)	0.200
Curvature	45 (19.48)	48 (20.78)	0.817	40 (17.32)	28 (12.12)	0.149	27 (11.69)	29 (12.55)	0.887

This table presents the clinical profiles of the two groups, stratified separately for each baseline global feature based on the median values. Continuous variables, including age and body mass index, are expressed as mean ± standard deviation and compared between the groups using an independent t-test. Categorical variables, including sex (male), hypertension, diabetes mellitus, dyslipidemia, current smoking, coronary artery disease, and atrial fibrillation, are presented as counts (percentages) and compared using the chi-square test.

Supplementary Table 6. Cox proportional hazards analysis results for the 10-year incidence of ischemic stroke based on the z-scores of chunk features adjusted for sex and age in the 462 patients (p-value < 0.1)

Arterial features	Baseline			Annual difference		
	Chunk	HR (95% CI)	p-value	Chunk	HR (95% CI)	p-value
Minimum-maximum diameter ratio	Rt ICA	1.33 (1.05–1.69)	0.020	Lt Cbll	0.82 (0.69–0.97)	0.020
	Rt basal ACA	1.16 (0.99–1.36)	0.073	Lt basal PCA	0.84 (0.72–0.98)	0.027
	Rt pial MCA	1.41 (1.19–1.68)	<0.001			
	Lt pial MCA	1.47 (1.23–1.76)	<0.001			
	Rt pial ACA	1.29 (1.08–1.53)	0.006			
	Lt pial ACA	1.39 (1.16–1.67)	<0.001			
	Lt Cbll	1.48 (1.19–1.85)	<0.001			
	Rt basal PCA	1.18 (1.01–1.39)	0.042			
	Lt basal PCA	1.50 (1.28–1.77)	<0.001			
Luminal circularity	Rt ICA	1.41 (0.99–2.01)	0.059	Rt pial ACA	1.14 (0.98–1.33)	0.091
	Lt ICA	1.35 (0.95–1.92)	0.095	Lt Cbll	0.84 (0.72–0.99)	0.032
	Rt pial MCA	1.39 (1.11–1.73)	0.004	Lt basal PCA	0.81 (0.71–0.91)	<0.001
	Lt pial MCA	1.35 (0.99–1.85)	0.055			
	Lt pial ACA	1.17 (0.97–1.40)	0.100			
	Lt Cbll	1.41 (1.08–1.85)	0.012			
	Lt basal PCA	2.24 (1.59–3.16)	<0.001			
Area	Rt pial MCA	0.86 (0.75–0.99)	0.034	Lt basal ACA	1.16 (1.02–1.32)	0.024
	Lt pial MCA	0.85 (0.72–1.00)	0.056	Lt pial MCA	1.12 (0.99–1.28)	0.077
	Lt VA	1.15 (1.01–1.31)	0.041	Rt pial ACA	0.85 (0.73–0.99)	0.031
				Rt VA	0.77 (0.65–0.92)	0.004
				Lt basal PCA	1.16 (0.98–1.36)	0.079
Perimeter	Rt pial MCA	0.87 (0.76–0.99)	0.033	Rt ICA	0.85 (0.71–1.01)	0.063
	Lt pial MCA	0.85 (0.72–1.02)	0.081	Lt basal ACA	1.14 (1.01–1.29)	0.040
	Lt VA	1.12 (0.99–1.28)	0.074	Rt pial ACA	0.85 (0.74–0.98)	0.020
	Lt Cbll	0.82 (0.64–1.04)	0.099	Rt VA	0.80 (0.68–0.94)	0.006
				Lt basal PCA	1.17 (0.99–1.38)	0.065
Maximum diameter	Rt pial MCA	0.85 (0.74–0.97)	0.019	Rt ICA	0.84 (0.70–1.01)	0.068
	Lt pial MCA	0.82 (0.69–0.98)	0.032	Lt basal ACA	1.12 (0.98–1.28)	0.098
	Lt VA	1.13 (0.99–1.29)	0.066	Rt pial MCA	1.12 (1.01–1.24)	0.026
	Lt basal PCA	0.86 (0.73–1.01)	0.059	Lt pial MCA	1.14 (0.98–1.33)	0.085
				Rt pial ACA	0.82 (0.68–0.99)	0.034
				Rt VA	0.80 (0.68–0.96)	0.014
				Lt basal PCA	1.19 (1.00–1.40)	0.045
Minimum diameter	Lt pial MCA	0.88 (0.78–1.01)	0.064	Rt ICA	0.86 (0.73–1.02)	0.084
	Lt VA	1.16 (0.99–1.36)	0.063	Lt basal ACA	1.15 (1.03–1.29)	0.012
				Rt pial MCA	1.11 (1.01–1.22)	0.023
				Lt pial MCA	1.16 (1.01–1.33)	0.042
				Rt pial ACA	0.85 (0.71–1.02)	0.077
				Rt VA	0.75 (0.58–0.97)	0.030
Maximum inscribed sphere radius	Lt pial MCA	0.89 (0.78–1.00)	0.056	Rt ICA	0.86 (0.72–1.03)	0.098
	Lt VA	1.15 (0.98–1.34)	0.082	Lt basal ACA	1.21 (0.99–1.47)	0.057
				Rt pial MCA	1.13 (1.01–1.26)	0.027
				Lt pial MCA	1.16 (1.01–1.33)	0.035
				Rt VA	0.75 (0.57–0.97)	0.030
Curvature	Rt ICA	0.71 (0.51–0.99)	0.045	Lt basal MCA	1.31 (1.09–1.57)	0.004
	Lt basal MCA	0.77 (0.66–0.90)	0.001	Rt pial ACA	0.84 (0.68–1.03)	0.093
	Lt pial MCA	0.69 (0.59–0.81)	<0.001			
	Lt pial ACA	0.77 (0.64–0.93)	0.006			
	BA	5.56×10^{-4} (4.66×10^{-5}–0.01)	<0.001			
	Rt Cbll	0.43 (0.23–0.82)	0.011			

Lt Cbll	0.79 (0.66–0.95)	0.010
Rt basal PCA	0.76 (0.64–0.90)	0.002
Lt basal PCA	0.77 (0.67–0.88)	<0.001

HR, hazard ratio; CI, confidence interval; Rt, Right; Lt, Left; ICA, internal carotid artery; MCA, middle cerebral artery; ACA, anterior cerebral artery; VA, vertebral artery; BA, basilar artery; Cbll, cerebellar artery; PCA, posterior cerebral artery.

Supplementary Table 7. Cox proportional hazards analysis results for the 10-year incidence of ischemic stroke, based on the z-scores of chunk features, adjusted for sex and age, in 245 patients with ICAS-positive. (p-value < 0.1)

Arterial features	Baseline			Annual difference		
	Chunk	HR (95% CI)	p-value	Chunk	HR (95% CI)	p-value
Minimum-maximum diameter ratio	Rt pial ACA	1.27 (0.99–1.64)	0.057	Rt basal PCA	1.28 (1.00–1.62)	0.046
	Lt pial ACA	1.21 (0.97–1.51)	0.092	Lt basal PCA	0.81 (0.66–1.00)	0.050
	Lt CbII	1.32 (1.02–1.70)	0.032			
	Lt basal PCA	1.33 (1.05–1.69)	0.016			
Luminal circularity	Rt VA	0.82 (0.65–1.04)	0.095	Lt basal ACA	0.82 (0.69–0.99)	0.042
	Lt CbII	1.33 (0.97–1.83)	0.075	Rt VA	1.29 (0.98–1.68)	0.067
	Lt basal PCA	2.08 (1.15–3.76)	0.015	Lt basal PCA	0.75 (0.64–0.88)	<0.001
Area				Rt ICA	0.73 (0.58–0.92)	0.007
				Lt basal MCA	0.87 (0.74–1.02)	0.079
				Lt basal ACA	1.20 (1.02–1.41)	0.028
				Rt VA	0.78 (0.58–1.03)	0.083
				Lt basal PCA	1.26 (1.01–1.57)	0.036
Perimeter				Rt ICA	0.67 (0.53–0.86)	0.001
				Lt basal MCA	0.86 (0.74–1.01)	0.058
				Lt basal ACA	1.19 (1.02–1.39)	0.025
				Rt VA	0.76 (0.59–0.99)	0.040
				Lt basal PCA	1.30 (1.02–1.64)	0.031
Maximum diameter				Rt ICA	0.67 (0.51–0.86)	0.002
				Lt basal MCA	0.88 (0.75–1.02)	0.088
				Lt basal ACA	1.16 (0.99–1.36)	0.069
				Rt VA	0.77 (0.58–1.01)	0.064
				Lt basal PCA	1.28 (1.03–1.59)	0.025
Minimum diameter				Rt ICA	0.75 (0.61–0.93)	0.008
Maximum inscribed sphere radius				Rt ICA	0.75 (0.59–0.95)	0.017
				Lt pial MCA	1.17 (0.97–1.41)	0.094
Curvature	Lt basal MCA	0.79 (0.61–1.01)	0.062	Lt basal MCA	1.39 (1.07–1.81)	0.014
	Rt basal ACA	0.69 (0.48–0.99)	0.044	Rt pial PCA	0.68 (0.45–1.04)	0.074
	Lt basal ACA	0.77 (0.62–0.96)	0.022			
	Rt pial MCA	0.81 (0.66–1.01)	0.060			
	Lt pial MCA	0.73 (0.58–0.90)	0.004			
	Lt pial ACA	0.82 (0.66–1.03)	0.090			
	Lt basal PCA	0.74 (0.60–0.92)	0.007			

ICAS, intracranial atherosclerotic stenosis; HR, hazard ratio; CI, confidence interval; Rt, Right; Lt, Left; ICA, internal carotid artery; MCA, middle cerebral artery; ACA, anterior cerebral artery; VA, vertebral artery; BA, basilar artery; CbII, cerebellar artery; PCA, posterior cerebral artery.

Supplementary Table 8. Cox proportional hazards analysis results for the 10-year incidence of ischemic stroke, based on the z-scores of chunk features, adjusted for sex and age, in 217 patients with ICAS-negative. (p-value < 0.1).

Arterial features	Baseline			Annual difference		
	Chunk	HR (95% CI)	p-value	Chunk	HR (95% CI)	p-value
Minimum-maximum diameter ratio	Rt ICA	1.78 (1.11–2.85)	0.017	Lt basal MCA	0.77 (0.61–0.98)	0.034
	Lt basal MCA	1.46 (1.13–1.89)	0.003	Rt basal ACA	0.77 (0.61–0.98)	0.036
	Rt pial MCA	1.73 (1.33–2.23)	<0.001	Lt pial MCA	0.76 (0.60–0.97)	0.026
	Lt pial MCA	1.85 (1.43–2.39)	<0.001	Rt VA	0.76 (0.55–1.05)	0.091
	Rt pial ACA	1.28 (0.99–1.64)	0.059	Lt Cbll	0.77 (0.58–1.02)	0.070
	Lt pial ACA	1.55 (1.16–2.07)	0.003			
	BA	1.35 (1.01–1.80)	0.040			
	Rt Cbll	1.27 (0.99–1.64)	0.061			
	Lt Cbll	1.85 (1.26–2.69)	0.001			
	Rt basal PCA	1.42 (1.14–1.78)	0.002			
	Lt basal PCA	1.68 (1.34–2.10)	<0.001			
Luminal circularity	Rt ICA	3.96 (1.82–8.64)	<0.001	Lt basal MCA	0.74 (0.58–0.96)	0.021
	Lt ICA	1.87 (1.26–2.78)	0.002	Lt pial MCA	0.64 (0.47–0.86)	0.003
	Lt basal MCA	1.49 (1.09–2.04)	0.012	Rt pial ACA	1.37 (1.03–1.82)	0.031
	Rt pial MCA	1.53 (1.13–2.06)	0.006	Rt VA	0.75 (0.55–1.01)	0.060
	Lt pial MCA	2.04 (1.41–2.95)	<0.001	Lt Cbll	0.79 (0.61–1.04)	0.095
	Lt VA	0.86 (0.72–1.02)	0.087			
	BA	1.61 (1.04–2.48)	0.032			
	Lt Cbll	1.62 (0.99–2.66)	0.055			
	Rt basal PCA	1.85 (1.36–2.52)	<0.001			
	Lt basal PCA	2.20 (1.48–3.28)	<0.001			
Area	Rt pial MCA	0.84 (0.70–1.01)	0.068	Rt pial MCA	1.20 (0.98–1.48)	0.084
	Lt pial MCA	0.79 (0.65–0.96)	0.017	Rt pial ACA	0.72 (0.54–0.96)	0.023
	Lt VA	1.29 (1.09–1.53)	0.003	Rt VA	0.69 (0.49–0.98)	0.035
Perimeter	Rt pial MCA	0.84 (0.70–1.00)	0.055	Rt pial MCA	1.22 (0.98–1.52)	0.074
	Lt pial MCA	0.79 (0.66–0.96)	0.015	Rt pial ACA	0.68 (0.52–0.89)	0.005
	Lt VA	1.25 (1.06–1.46)	0.006			
Maximum diameter	Rt pial MCA	0.81 (0.67–0.98)	0.032	Rt pial MCA	1.17 (1.03–1.33)	0.015
	Lt pial MCA	0.76 (0.62–0.93)	0.007	Lt pial MCA	1.17 (0.97–1.42)	0.098
	Lt VA	1.24 (1.06–1.45)	0.006	Rt pial ACA	0.72 (0.53–0.97)	0.031
	Lt basal PCA	0.84 (0.69–1.02)	0.073			
Minimum diameter	Lt VA	1.22 (0.97–1.53)	0.087	Lt basal ACA	1.42 (1.06–1.91)	0.018
				Rt pial MCA	1.16 (1.03–1.32)	0.019
				Rt VA	0.64 (0.45–0.92)	0.015
Maximum inscribed sphere radius				Lt basal ACA	1.44 (1.08–1.93)	0.013
				Rt pial MCA	1.18 (1.04–1.34)	0.009
				Rt VA	0.63 (0.43–0.92)	0.017
Curvature	Rt ICA	0.69 (0.52–0.92)	0.010	Lt pial MCA	1.30 (1.05–1.61)	0.017
	Lt basal MCA	0.72 (0.58–0.89)	0.003	Rt pial ACA	0.73 (0.58–0.92)	0.008
	Lt pial MCA	0.68 (0.55–0.83)	<0.001	Rt Cbll	4.46 (0.83–23.78)	0.080
	Lt pial ACA	0.72 (0.54–0.97)	0.030			
	Rt Cbll	0.19 (0.05–0.70)	0.013			
	Lt Cbll	0.74 (0.58–0.93)	0.011			
	Rt basal PCA	0.68 (0.53–0.87)	0.002			
	Lt basal PCA	0.75 (0.63–0.90)	0.002			

ICAS, intracranial atherosclerotic stenosis; HR, hazard ratio; CI, confidence interval; Rt, Right; Lt, Left; ICA, internal carotid artery; MCA, middle cerebral artery; ACA, anterior cerebral artery; VA, vertebral artery; BA, basilar artery; Cbll, cerebellar artery; PCA, posterior cerebral artery.

Supplementary Table 9. Cox proportional hazards analysis results for the 10-year incidence of ischemic stroke, based on the z-scores of chunk features, adjusted for sex and age, in 271 patients with stroke/TIA indexed baseline. (p-value < 0.1).

Arterial Features	Baseline			Annual difference		
	Chunk	HR (95% CI)	p-value	Chunk	HR (95% CI)	p-value
Minimum-maximum diameter ratio	Rt pial MCA	1.21 (0.97 - 1.52)	0.095	Lt basal MCA	0.78 (0.61 - 1.00)	0.055
	Lt pial MCA	1.34 (1.01 - 1.79)	0.046	Lt pial ACA	0.80 (0.61 - 1.04)	0.092
	Lt pial ACA	1.57 (1.16 - 2.10)	0.003	Lt basal PCA	0.77 (0.63 - 0.94)	0.011
	Lt basal PCA	1.56 (1.19 - 2.05)	0.001			
Luminal circularity	Lt pial ACA	1.28 (0.98 - 1.69)	0.072	Lt basal MCA	0.75 (0.57 - 0.99)	0.044
	Lt basal PCA	2.02 (1.14 - 3.57)	0.015	Rt pial ACA	1.19 (0.99 - 1.44)	0.064
				Lt VA	0.58 (0.37 - 0.89)	0.013
				BA	0.57 (0.35 - 0.94)	0.026
				Lt basal PCA	0.74 (0.62 - 0.88)	<0.001
Area	Rt pial ACA	1.16 (1.00 - 1.34)	0.057	Lt basal ACA	1.22 (1.03 - 1.44)	0.018
				Rt pial ACA	0.83 (0.68 - 1.01)	0.068
				Rt VA	0.71 (0.50 - 0.99)	0.043
				BA	2.08 (0.95 - 4.59)	0.068
				Lt basal PCA	1.30 (1.04 - 1.62)	0.022
Perimeter	Lt basal MCA	0.83 (0.67 - 1.03)	0.095	Lt basal ACA	1.21 (1.03 - 1.41)	0.022
	Rt pial ACA	1.14 (0.99 - 1.30)	0.068	Rt pial ACA	0.85 (0.71 - 1.01)	0.060
				Rt VA	0.74 (0.54 - 1.01)	0.061
				BA	2.29 (1.09 - 4.81)	0.028
				Lt basal PCA	1.32 (1.03 - 1.70)	0.028
Maximum diameter	Rt pial ACA	1.20 (1.00 - 1.46)	0.054	Lt basal ACA	1.17 (0.99 - 1.39)	0.063
				Rt pial MCA	1.16 (1.00 - 1.33)	0.049
				Rt pial ACA	0.77 (0.61 - 0.99)	0.039
				Rt VA	0.72 (0.51 - 1.01)	0.058
				BA	2.30 (1.00 - 5.28)	0.050
				Lt basal PCA	1.32 (1.05 - 1.66)	0.019
Minimum diameter	Lt pial MCA	0.83 (0.67 - 1.02)	0.079	Lt basal ACA	1.20 (1.03 - 1.41)	0.021
	Rt pial ACA	1.21 (1.00 - 1.45)	0.045	Rt pial MCA	1.16 (1.01 - 1.32)	0.035
Maximum inscribed sphere radius	Rt basal MCA	1.23 (0.97 - 1.56)	0.088	Rt pial MCA	1.16 (0.98 - 1.37)	0.078
	Lt pial MCA	0.82 (0.68 - 1.00)	0.047			
	Rt VA	0.78 (0.59 - 1.04)	0.089			
	Rt Cbll	0.83 (0.68 - 1.02)	0.072			
Curvature	Lt basal MCA	0.76 (0.60 - 0.96)	0.024	Lt basal MCA	1.36 (1.06 - 1.76)	0.017
	Rt pial MCA	0.83 (0.68 - 1.02)	0.075	Rt pial ACA	0.70 (0.57 - 0.88)	0.002
	Lt pial MCA	0.63 (0.50 - 0.79)	<0.001	Lt VA	1.28 (1.02 - 1.62)	0.034
	Rt pial ACA	1.24 (0.98 - 1.57)	0.070	BA	2.12 (1.41 - 3.19)	<0.001
	Lt pial ACA	0.74 (0.57 - 0.95)	0.020			
	Rt VA	1.27 (1.01 - 1.59)	0.042			
	BA	4.07×10^{-11} (3.73×10^{-14} - 4.45×10^{-8})	<0.001			
	Lt basal PCA	0.81 (0.67 - 0.98)	0.031			

HR, hazard ratio; CI, confidence interval; Rt, Right; Lt, Left; ICA, internal carotid artery; MCA, middle cerebral artery; ACA, anterior cerebral artery; VA, vertebral artery; BA, basilar artery; Cbll, cerebellar artery; PCA, posterior cerebral artery.

Supplementary Table 10. Cox proportional hazards analysis results for the 10-year incidence of ischemic stroke, based on the z-scores of chunk features, adjusted for sex and age, in 191 patients with non-stroke/TIA indexed baseline. (p-value < 0.1).

Arterial Features	Baseline			Annual difference		
	Chunk	HR(95% CI)	p-value	Chunk	HR(95% CI)	p-value
Minimum-maximum diameter ratio	Rt ICA	1.32 (1.04 - 1.68)	0.021	Lt Cbll	0.77 (0.63 - 0.94)	0.012
	Rt basal ACA	1.22 (0.98 - 1.51)	0.076			
	Rt pial MCA	1.66 (1.30 - 2.13)	<0.001			
	Lt pial MCA	1.59 (1.26 - 2.00)	<0.001			
	Rt pial ACA	1.64 (1.27 - 2.11)	<0.001			
	Lt pial ACA	1.32 (1.03 - 1.69)	0.026			
	Rt Cbll	1.21 (0.98 - 1.50)	0.072			
	Lt Cbll	1.64 (1.23 - 2.18)	<0.001			
	Rt basal PCA	1.27 (1.03 - 1.57)	0.028			
	Lt basal PCA	1.48 (1.21 - 1.82)	<0.001			
	Rt pial PCA	1.25 (1.00 - 1.57)	0.054			
	Lt pial PCA	1.40 (0.98 - 2.02)	0.066			
Luminal circularity	Rt ICA	1.42 (1.00 - 2.00)	0.048	Lt Cbll	0.87 (0.74 - 1.01)	0.070
	Lt ICA	1.45 (1.15 - 1.84)	0.002			
	Rt pial MCA	1.76 (1.25 - 2.47)	0.001			
	Lt pial MCA	1.50 (1.14 - 1.97)	0.004			
	Rt pial ACA	1.47 (1.13 - 1.90)	0.003			
	Lt Cbll	1.63 (1.13 - 2.35)	0.009			
	Lt basal PCA	1.95 (1.42 - 2.68)	<0.001			
	Rt pial PCA	1.30 (0.99 - 1.72)	0.062			
Area	Rt pial MCA	0.82 (0.67 - 1.00)	0.056	Rt ICA	0.78 (0.62 - 0.98)	0.036
	Rt pial ACA	0.82 (0.66 - 1.01)	0.065	Lt basal MCA	0.87 (0.75 - 1.02)	0.079
	Lt VA	1.29 (1.09 - 1.53)	0.003	Rt pial MCA	1.22 (0.99 - 1.51)	0.061
				Rt VA	0.81 (0.63 - 1.03)	0.080
Perimeter	Rt pial MCA	0.83 (0.68 - 1.00)	0.051	Rt ICA	0.77 (0.61 - 0.96)	0.022
	Rt pial ACA	0.80 (0.65 - 0.99)	0.043	Lt basal MCA	0.88 (0.76 - 1.02)	0.084
	Lt VA	1.23 (1.04 - 1.44)	0.013	Rt pial MCA	1.23 (0.99 - 1.53)	0.060
	Lt Cbll	0.81 (0.64 - 1.03)	0.083	Rt VA	0.81 (0.65 - 1.02)	0.076
Maximum diameter	Rt pial MCA	0.81 (0.66 - 0.98)	0.032	Rt ICA	0.77 (0.61 - 0.96)	0.020
	Lt pial MCA	0.84 (0.70 - 1.01)	0.063	Rt pial MCA	1.23 (0.99 - 1.54)	0.067
	Rt pial ACA	0.77 (0.62 - 0.96)	0.020			
	Lt VA	1.23 (1.04 - 1.45)	0.015			
Minimum diameter	Lt VA	1.28 (1.03 - 1.59)	0.023	Rt ICA	0.77 (0.61 - 0.97)	0.029
				Rt pial MCA	1.23 (1.00 - 1.51)	0.049
				Lt pial MCA	1.19 (0.98 - 1.44)	0.077
Maximum inscribed sphere radius	Lt VA	1.26 (1.02 - 1.56)	0.032	Rt ICA	0.76 (0.60 - 0.95)	0.018
				Lt basal ACA	1.35 (1.06 - 1.73)	0.015
				Rt pial MCA	1.22 (1.00 - 1.49)	0.054
				Lt pial MCA	1.20 (0.99 - 1.45)	0.065
Curvature	Rt ICA	0.76 (0.63 - 0.93)	0.007	Rt ICA	1.23 (0.99 - 1.54)	0.065
	Lt ICA	0.76 (0.63 - 0.91)	0.004	Lt ICA	1.21 (1.02 - 1.45)	0.033
	Lt basal MCA	0.76 (0.61 - 0.95)	0.015	Rt basal MCA	1.24 (0.99 - 1.56)	0.065
	Lt pial MCA	0.72 (0.59 - 0.89)	0.002	Lt basal MCA	1.25 (0.96 - 1.62)	0.096
	Rt pial ACA	0.71 (0.57 - 0.89)	0.003	Lt pial ACA	0.87 (0.75 - 1.00)	0.055
	Rt Cbll	0.74 (0.61 - 0.89)	0.002			
	Lt Cbll	0.69 (0.54 - 0.88)	0.003			
	Rt basal PCA	0.72 (0.57 - 0.90)	0.004			
	Lt basal PCA	0.72 (0.59 - 0.88)	0.001			
	Rt pial PCA	0.80 (0.63 - 1.01)	0.056			

HR, hazard ratio; CI, confidence interval; Rt, Right; Lt, Left; ICA, internal carotid artery; MCA, middle cerebral artery; ACA, anterior cerebral artery; VA, vertebral artery; BA, basilar artery; Cbll, cerebellar artery; PCA, posterior cerebral artery.

References

1. Hong SW, Song HN, Choi JU*, et al*. Automated in-depth cerebral arterial labelling using cerebrovascular vasculature reframing and deep neural networks. *Sci Rep*. 2023;13(1):3255.
