# Supplementary material for: Association of cerebrovascular morphology with ischaemic stroke considering intracranial stenosis
Source: Brain Commun. 2026 Feb 10;8(2):fcag037. doi: 10.1093/braincomms/fcag037 (PMC13044395; doi:10.1093/braincomms/fcag037)
Supplement: fcag037_Supplementary_Data [file fcag037_supplementary_data.zip › Supplementary_materials_python_code.pdf]

Python code for Figure 2, Supplementary Figures 3 and 4

The following code illustrates how to generate a Kaplan–Meier survival plot using the global feature "baseline minimum–maximum diameter ratio"

```
# ----------------------------------------------------------
# Purpose:
#     Generate Kaplan–Meier survival curves for a baseline global feature,
#     dichotomized at the median, and compare groups using the log-rank test.
#
# Study design:
#     - Input data: entire_final.csv
#     - Exclusion filter: Hemo1_Aneu2 == 0
#     - Time-to-event column: new_event_duration
#     - Event indicator: new_ischemic_stroke_event (1 = event, 0 = censored)
#
# Group definition:
#     - Predictor: Before_MinMaxDiameterRatio (baseline minimum-maximum diameter ratio value)
#     - High vs. Low defined by the median of Before_MinMaxDiameterRatio
#
# Modeling details:
#     - Kaplan–Meier estimators are fit separately in each group.
#     - Two-sided log-rank test compares survival distributions.
#
# Output:
#     - Survival curves with 95% CIs and group-specific labels (n).
#     - Reported p-value from the log-rank test on the main plot.
#     - A compact "number at risk (censored)" panel at predefined time points.
# ----------------------------------------------------------

import pandas as pd
import matplotlib.pyplot as plt
from lifelines import KaplanMeierFitter
from lifelines.statistics import logrank_test
import numpy as np

# 1) Load dataset and apply study filter
#       Keep only participants meeting the study inclusion (exclude prior ICH/SAH, unruptured aneurysm).
data = pd.read_csv("entire_final.csv")                    # Input CSV (global features successfully extracted)
data = data[(data['Hemo1_Aneu2'] == 0)]                   # Apply exclusion criteria

# 2) Define grouping variable (median split of selected feature)
#       Use the baseline perimeter to dichotomize subjects; record group sizes for legend labels.
column_select = data['Before_MinMaxDiameterRatio']
median_value = column_select.median(skipna=True)
group_high = data[column_select >= median_value]      # High group (≥ median)
group_low   = data[column_select <   median_value]      # Low group    (< median)
count_high = len(group_high)
count_low   = len(group_low)

# 3) Initialize Kaplan–Meier estimators for both groups
#       Separate estimators allow independent survival function estimation.
kmf_high = KaplanMeierFitter()
kmf_low   = KaplanMeierFitter()

# 4) Fit survival curves to high and low groups
#       Label includes group name and sample size (n) for transparency.
label_name = 'MMR$_{base}$'
kmf_high.fit(
```

```
        group_high['new_event_duration'],
        event_observed=group_high['new_ischemic_stroke_event'],
        label='High ' + label_name + ', n=' + str(count_high)
)
kmf_low.fit(
        group_low['new_event_duration'],
        event_observed=group_low['new_ischemic_stroke_event'],
        label='Low ' + label_name + ', n=' + str(count_low)
)

# 5) Perform log-rank test to compare survival distributions
#       Two-sample log-rank test; store p-value for annotation.
results = logrank_test(
        group_high['new_event_duration'],
        group_low['new_event_duration'],
        event_observed_A=group_high['new_ischemic_stroke_event'],
        event_observed_B=group_low['new_ischemic_stroke_event']
)
p_value = results.p_value

# 6) Plot Kaplan–Meier survival functions
#       Show 95% CIs; use contrasting colors for readability.
plt.figure(figsize=(9.5, 6))
kmf_high.plot_survival_function(ci_show=True, color='tomato')
kmf_low.plot_survival_function(ci_show=True,    color='dodgerblue')

# Axes, labels, and grid styling (journal-friendly)
plt.xlabel('Timeline (Days)', fontsize=18)
plt.ylabel('Survival Probability', fontsize=18)
plt.grid(False)

# Axis customization (ticks and limits tailored to follow-up range)
plt.xticks(fontsize=13)
plt.yticks(fontsize=13)
plt.xlim(-200, 3650)      # allow left padding to avoid clipping labels
plt.ylim(-0.05, 1.02)    # slight margins beyond [0,1] for aesthetics

# Legend: place inside plot with compact offset; include group sizes
plt.legend(loc='lower left', bbox_to_anchor=(0.005, 0.01), fontsize=18)

# Display formatted p-value (with thresholding for small values)
if p_value < 0.001:
        p_value_text = 'p-value: <0.001'
else:
        p_value_text = f'p-value: {p_value:.3f}'
plt.text(0, 0.25, p_value_text, fontsize=18)

# Clean presentation: remove top/right spines (reduces visual clutter)
ax = plt.gca()
ax.spines['top'].set_visible(False)
ax.spines['right'].set_visible(False)

# 7) Define custom time points for risk table annotation
#       These should reflect clinically relevant intervals and follow-up length.
time_points = [0, 500, 1000, 1500, 2000, 2500, 3000, 3500]

# Compute cumulative counts up to each time point:
# - events_*: number of observed events
# - censored_*: number censored
```

```
# - at_risk_*: remaining at risk (= N - events - censored)
events_high     = [sum((group_high['new_ischemic_stroke_event'] == 1) &
                        (group_high['new_event_duration'] <= t)) for t in time_points]
censored_high = [sum((group_high['new_ischemic_stroke_event'] == 0) &
                        (group_high['new_event_duration'] <= t)) for t in time_points]
at_risk_high    = [len(group_high) - censored_high[i] - events_high[i] for i in range(len(time_points))]

events_low       = [sum((group_low['new_ischemic_stroke_event'] == 1) &
                        (group_low['new_event_duration'] <= t)) for t in time_points]
censored_low    = [sum((group_low['new_ischemic_stroke_event'] == 0) &
                        (group_low['new_event_duration'] <= t)) for t in time_points]
at_risk_low     = [len(group_low) - censored_low[i] - events_low[i]    for i in range(len(time_points))]

# 8) Add separate risk table panel (number at risk and censored)
#       Compact strip beneath the main plot: shows "at-risk(censored)" per time point.
fig, ax1 = plt.subplots(figsize=(9.5, 1))
ax1.set_yticks([60, 45])
ax1.set_yticklabels(['                        ','                        '])    # hide y tick labels while preserving spacing

for i, t in enumerate(time_points):
    # High group annotation (row above)
    ax1.text(t+50, 57, f'{at_risk_high[i]}({censored_high[i]})',
             fontsize=13, ha='center', color='black', fontfamily='monospace')
    # Low group annotation (row below)
    ax1.text(t+50, 42, f'{at_risk_low[i]}({censored_low[i]})',
             fontsize=13, ha='center', color='black', fontfamily='monospace')

ax1.set_title('Number at risk (censored)', fontsize=13, loc='left')
ax1.set_ylim(35, 65)

# Color keys to visually link rows to curves above
ax1.add_patch(plt.Rectangle((-400, 54), 200, 10, color='tomato',       clip_on=False))    # High
ax1.add_patch(plt.Rectangle((-400, 39), 200, 10, color='dodgerblue', clip_on=False)) # Low

# Clean axis formatting for the risk table
plt.xlim(-200, 3650)
ax1.spines['top'].set_visible(False)
ax1.spines['right'].set_visible(False)
ax1.yaxis.set_ticks_position('none')
ax1.tick_params(axis='x', labelsize=13)

# 9) Display final survival plot with risk table
plt.show()
```

Python code for Figure 3, Supplementary Figure 5

The following code exemplifies the analysis applied to the total group (n = 462) presented in Figure 3. Subgroup-specific datasets were then constructed according to prespecified criteria, and the same analytical pipeline was applied to generate the remaining figure panels

```
# -----------------------------------------------------------
# Purpose:
#     Run chunk-level Cox proportional hazards analyses for selected
#     morphological feature families (baseline and annualized change),
#     and visualize the spatial distribution of effects on a 3D template.
#
# Study design:
#     - Input data: chunk_final.csv
#     - Exclusion filter: Hemo1_Aneu2 == 0
#     - Time-to-event column: new_event_duration
#     - Event indicator: new_ischemic_stroke_event (1 = event, 0 = censored)
#     - Adjustment covariates: before_age, Sex
#
# Feature families:
#     - "Before_*"    → baseline values per chunk
#     - "*difference_per_year" → annualized longitudinal change per chunk
#
# Modeling details:
#     - For each feature column, fit a CoxPH model with the feature (z-scored),
#        adjusting for before_age and Sex (unscaled).
#     - Drop rows with missing values in analysis columns prior to fitting.
#
# Output:
#     - total_result_df: Long-form table of per-feature Cox results
#     - total_result_df_fin: Adds HR override by p-value and log(HR)
#     - 3D scatter panels: color encodes log(HR), point size ∝ area
# -----------------------------------------------------------

import pandas as pd
import numpy as np
from lifelines import CoxPHFitter
import matplotlib.pyplot as plt

# 1) Load dataset and apply study filter
df = pd.read_csv('chunk_final.csv')
df = df[(df['Hemo1_Aneu2'] == 0)]
print(len(df))    # Sanity check: sample size after filtering

# 2) Build feature families by column name patterns
#       - Families are lists of feature columns that share semantics.
MMR_before           = [col for col in df.columns if 'Before_' in col and 'MinMaxDiameterRatio' in col]
LC_before            = [col for col in df.columns if 'Before_' in col and 'LuminalCircularity' in col]
Area_before          = [col for col in df.columns if 'Before_' in col and 'Area' in col]
Perimeter_before   = [col for col in df.columns if 'Before_' in col and 'Perimeter' in col]
MinD_before          = [col for col in df.columns if 'Before_' in col and 'MinDiameter' in col]
MaxD_before          = [col for col in df.columns if 'Before_' in col and 'MaxDiameter' in col and 
'MinMaxDiameter' not in col]
MISR_before          = [col for col in df.columns if 'Before_' in col and 'MaxInscribedSphereR' in col]
Curvature_before   = [col for col in df.columns if 'Before_' in col and 'Curvature' in col]

MMR_diff                    = [col for col in df.columns if 'difference_per_year' in col and 
'MinMaxDiameterRatio' in col]
LC_diff               = [col for col in df.columns if 'difference_per_year' in col and 'LuminalCircularity' in 
col]
```

```
Area_diff         = [col for col in df.columns if 'difference_per_year' in col and 'Area' in col]
Perimeter_diff    = [col for col in df.columns if 'difference_per_year' in col and 'Perimeter' in col]
MinD_diff         = [col for col in df.columns if 'difference_per_year' in col and 'MinDiameter' in col]
MaxD_diff         = [col for col in df.columns if 'difference_per_year' in col and 'MaxDiameter' in col
and 'MinMaxDiameter' not in col]
MISR_diff         = [col for col in df.columns if 'difference_per_year' in col and 'MaxInscribedSphereR'
in col]
Curvature_diff    = [col for col in df.columns if 'difference_per_year' in col and 'Curvature' in col]

length_before     = [col for col in df.columns if 'Before_' in col and 'length' in col]   # kept for
completeness

# 3) Choose which feature families to analyze in this run
all_interaction_target_columns = [MMR_before, Area_before, Curvature_before,
                                  MMR_diff,   Area_diff,   Curvature_diff]

# 4) Z-score normalization
def z_score_transform(df, columns):
    for col in columns:
        df[col] = (df[col] - df[col].mean()) / df[col].std(ddof=1)
    return df

# 5) Apply z-score to selected features (covariates remain on original scales)
df = z_score_transform(df, all_interaction_target_columns)   # as-is (will fail if not flattened)

# 6) Specify time-to-event, event indicator, and adjustment covariates
duration_col    = 'new_event_duration'
event_col       = 'new_ischemic_stroke_event'
adjustment_vars = ['before_age', 'Sex']

# 7) Prepare container for Cox results accumulated across features
total_result_df = pd.DataFrame()

# 8) Cox PH modeling loop
#    - For each feature family, fit a Cox model per feature:
#      Surv(time, event) ~ feature (z-scored) + before_age + Sex
for target_columns in all_interaction_target_columns:
    # Minimal analysis frame for current family
    cox_data = df[[duration_col, event_col] + adjustment_vars + target_columns]
    cph = CoxPHFitter()

    for col in target_columns:
        # Drop rows with NA across analysis columns for a clean fit
        temp_cox_df = cox_data[[duration_col, event_col, col] + adjustment_vars].dropna()
        cph.fit(temp_cox_df, duration_col=duration_col, event_col=event_col)

        # Extract per-feature summary (HR, CI, p, etc.) and append metadata
        summary = cph.summary
        temp_result_df = pd.DataFrame(summary.loc[col, :]).T
        temp_result_df['target_column'] = col
        total_result_df = pd.concat([total_result_df, temp_result_df])

# 9) Post-processing: override HR by p-value threshold and compute log(HR)
#    - If p >= 0.003125, set HR=1 → log(HR)=0 (for visualization de-emphasis)
def pvalue_select(dataframe):
    for index, row in dataframe.iterrows():
        if row['p'] >= 0.003125:
            dataframe.loc[index, 'HR'] = 1
        else:
```

```
            dataframe.loc[index, 'HR'] = row['exp(coef)']
    dataframe['log(HR)'] = dataframe['HR'].apply(lambda x: np.log(x))
    return dataframe

# 10) Derive 'Chunk' labels from feature names and finalize analysis table
#       - Assumes feature names follow pattern like "Before_<Chunk>_...".
total_result_df['Chunk'] = total_result_df['target_column'].apply(lambda x: x.split('_')[1])
total_result_df.reset_index(drop=True, inplace=True)
total_result_df_fin = pvalue_select(total_result_df)

# 11) Load 3D template for vascular spatial layout
#       - Must contain: Chunk, X, Y, Z, Area (for point sizing)
html_concat_data_fin_template = pd.read_csv('html_concat_data_fin_template.csv')

# 12) Wrap final table into a list for potential extension to multiple datasets
all_data_list = [total_result_df_fin]

from matplotlib import cm
from matplotlib.colors import Normalize

# 13) Configure figure and subplot indexing
fig = plt.figure(figsize=(28, 8))
i = 0   # subplot counter

# 14) Generate one 3D scatter panel per feature family
for using_data in all_data_list:
    using_data_modify = using_data[['Chunk', 'HR', 'target_column', 'log(HR)']]

    for using_target_column_list in all_interaction_target_columns:
        i += 1

        # Merge Cox results (current family) with 3D template by 'Chunk'
        temp_df_for_figure = using_data_modify[
            using_data_modify['target_column'].isin(using_target_column_list)
        ]
        df_for_figure = pd.merge(
            html_concat_data_fin_template,
            temp_df_for_figure,
            on='Chunk',
            how='left'
        )

        # 15) 3D scatter: color = log(HR), size ∝ Area (scaled), common color limits
        ax = plt.subplot(1, 6, i, projection='3d')
        ax.scatter(
            df_for_figure.X, df_for_figure.Y, df_for_figure.Z,
            c=df_for_figure['log(HR)'],
            cmap='coolwarm',
            s=df_for_figure.Area * 2,
            vmin=-0.8, vmax=0.8
        )

        # Minimalistic axes for a clean, manuscript-ready look
        ax.set_xlabel('X', fontsize=0)
        ax.set_ylabel('Y', fontsize=0)
        ax.set_zlabel('Z', fontsize=0)
        ax.view_init(20, 5)
        ax.xaxis.set_pane_color((1.0, 1.0, 1.0, 0.0))
        ax.yaxis.set_pane_color((1.0, 1.0, 1.0, 0.0))
```

```
        ax.zaxis.set_pane_color((1.0, 1.0, 1.0, 0.0))
        plt.rc('xtick', labelsize=0)    # hide x tick labels
        plt.rc('ytick', labelsize=0)    # hide y tick labels

# 16) Tighten layout and render the figure
plt.subplots_adjust(wspace=-0.15, hspace=-0.15)
plt.show()
```

Python code for Table 3

```
# ----------------------------------------------------------
# Purpose:
#     Generate a summary table of hazard ratios (HR), 95% confidence intervals,
#     and p-values for all global features, adjusted for prespecified covariates.
#
# Study design:
#     - Input data: entire_final.csv
#     - Exclusion filter: Hemo1_Aneu2 == 0
#     - Time-to-event column: new_event_duration
#     - Event indicator: new_ischemic_stroke_event (1 = event, 0 = censored)
#     - Adjustment covariates: before_age, Sex
#
# Feature selection:
#     - Candidate predictors are all columns whose names contain either
#        "Before_" → baseline value or "difference_per_year" → annualized change.
#
# Modeling details:
#     - For each predictor, we fit a Cox proportional hazards model including
#        the predictor (z-scored within the analysis subset) and the adjustment
#        covariates (kept on their original scales).
#     - Rows with missing values or infinite values in the analysis columns
#        are removed prior to fitting.
#     - Predictors with zero standard deviation (constant) are skipped.
#
# Output:
#     - A table with the following columns:
#           * Global features
#           * HR(95% CI): formatted HR with lower–upper 95% CI
#           * p-value: formatted p-value
#     - All predictors are reported regardless of p-value.
#     - The table is sorted by the raw HR (descending).
#     - Optionally displayed with basic styling (Jupyter) and copied to clipboard.
# ----------------------------------------------------------

import pandas as pd
from lifelines import CoxPHFitter

# 1) Paths and column names
CSV_PATH = 'entire_final.csv'                    # Input CSV file path (successfully extracted global feature data, n=500)
duration_col = 'new_event_duration'              # Time-to-event column
event_col = 'new_ischemic_stroke_event'          # Event indicator (1/0)
adjustment_vars = ['before_age', 'Sex']          # Covariates for adjustment


# 2) Load data and apply study filter
#     - Keep rows with Hemo1_Aneu2 == 0 as per study design. (excluded: history of ICH/SAH, unruptured aneurysm)
data = pd.read_csv(CSV_PATH)
data = data[(data['Hemo1_Aneu2'] == 0)]

# 3) Select candidate predictors
#     - Columns containing "Before_" or "difference_per_year"
columns_to_analyze = [
    c for c in data.columns
    if ("difference_per_year" in c) or ("Before_" in c)
]
```

```
# Cox model instance
cox_model = CoxPHFitter()

# 4) Formatting helpers
#     - Scientific notation for very small numbers (<0.01) with Unicode superscripts
def format_superscript(num: int) -> str:
    """Convert an integer exponent to Unicode superscripts for display."""
    superscript_map = {'0':'⁰','1':'¹','2':'²','3':'³','4':'⁴','5':'⁵','6':'⁶','7':'⁷','8':'⁸','9':'⁹','-':'⁻'}
    return ''.join(superscript_map.get(ch, ch) for ch in str(num))

def to_sci_text(x: float) -> str:
    """Return x in scientific notation with superscripts if x < 0.01; otherwise with 2 decimals."""
    if x < 0.01:
        base, exp = f"{x:.2e}".split('e')
        return f"{base} × 10{format_superscript(int(exp))}"
    return f"{x:.2f}"

def format_hr_ci(hr: float, ci_lower: float, ci_upper: float) -> str:
    """Format HR and 95% CI for reporting."""
    return f"{to_sci_text(hr)} ({to_sci_text(ci_lower)} - {to_sci_text(ci_upper)})"

def format_pvalue(p: float) -> str:
    """Format p-value with scientific notation and superscripts if < 0.01."""
    if p < 0.01:
        base, exp = f"{p:.2e}".split('e')
        return f"{base} × 10{format_superscript(int(exp))}"
    return f"{p:.3f}"

# 5) Fit Cox models per predictor and collect results
rows = []
for column in columns_to_analyze:
    # Exclude time/event/covariates from the predictor loop
    if column in [duration_col, event_col] + adjustment_vars:
        continue

    # Prepare analysis frame: predictor + covariates + time/event
    use_cols = [duration_col, event_col, column] + adjustment_vars
    df = (
        data[use_cols]
        .replace([float('inf'), float('-inf')], pd.NA)    # Convert infinities to NA
        .dropna()                                          # Drop rows with missing values
    )

    # Skip constant predictors (std = 0) or undefined std
    std = df[column].std(ddof=1)
    if pd.isna(std) or std == 0:
        continue

    # Z-score the predictor only; covariates remain unscaled
    df[column] = (df[column] - df[column].mean()) / std

    # Fit Cox proportional hazards model
    # lifelines uses all columns except duration/event as covariates
    cox_model.fit(df, duration_col=duration_col, event_col=event_col)
    summ = cox_model.summary

    # Extract statistics
    p_value = float(summ.loc[column, 'p'])
```

```
        hr = float(summ.loc[column, 'exp(coef)'])
        ci_lower = float(summ.loc[column, 'exp(coef) lower 95%'])
        ci_upper = float(summ.loc[column, 'exp(coef) upper 95%'])

        # Append unfiltered result row
        rows.append({
            'Global features': column,                    # Original predictor name
            'HR(95% CI)': format_hr_ci(hr, ci_lower, ci_upper),
            'p-value': format_pvalue(p_value),
            'hr_raw': hr                                  # For sorting only (not displayed)
        })

# 6) Build and present the table
#     - Sort by hr_raw in descending order
#     - Display styled table (optional) and copy to clipboard
result_df = pd.DataFrame(rows)
if not result_df.empty:
    result_df = result_df.sort_values('hr_raw', ascending=False).reset_index(drop=True)
    result_df = result_df[['Global features', 'HR(95% CI)', 'p-value']]

    # Optional: styled display (Jupyter)
    from IPython.display import display
    styled = (
        result_df.style
        .set_table_attributes('style="width:100%"')
        .set_properties(**{'background-color': 'white', 'color': 'black', 'text-align': 'left'})
        .set_table_styles([{'selector': 'th', 'props': [('text-align', 'left'), ('background-color', 'white')]}])
    )
    display(styled)

    # Optional: copy to clipboard (works in local environments)
    result_df.to_clipboard(index=False)
```

Python code for Supplementary Figure 1

```
# ----------------------------------------------------------
# Purpose:
#     Visualize the vascular 3D template with chunk-wise, manually
#     assigned colors to highlight arterial territories. Point size
#     scales with 'Area' to convey relative importance/extent.
#
# Data:
#     - Template file: html_concat_data_fin_template.csv
#     - Required columns: Chunk, X, Y, Z, Area
#
# Coloring scheme:
#     - A manual color map is defined for normalized chunk labels.
#     - Unspecified chunks fall back to a neutral default color.
#
# Output:
#     - 3D scatter plot: each chunk drawn at (X, Y, Z) with assigned color,
#        marker size ∝ Area. Axes/ticks minimized for a clean, manuscript-ready figure.
# ----------------------------------------------------------

import pandas as pd
from matplotlib import cm
from matplotlib.colors import to_hex
import matplotlib.pyplot as plt
import numpy as np
import re

# 1) Define a manual color palette for normalized chunk names
manual_color_map = {
  'LtICA': '#072B8C',     'RtICA': '#8A0213',
  'LtBasalMCA': '#319CFD',   'RtBasalMCA': '#F95C5C',
  'LtPialMCA':   '#2C42EA',   'RtPialMCA':   '#D00505',
  'LtBasalACA': '#45D4F9',
  'RtBasalACA': '#FF9380',
  'LtPialACA':   '#A5F6FF',
  'RtPialACA':   '#FFBEB8',
  'ACoA': '#000000'
}

# Alternative palette for posterior circulation (toggle as needed)
# manual_color_map = {
#        'LtVA': '#072B8C',     'RtVA' : '#8A0213',
#        'LtBasalPCA': '#319CFD',   'RtBasalPCA': '#F95C5C',
#        'LtPialPCA':   '#2C42EA',   'RtPialPCA':   '#D00505',
#        'LtCbll': '#45D4F9',   'RtCbll': '#FF9380',
#        'BA': '#000000'
# }

# 2) Load the 3D template containing chunk coordinates and sizes
html_concat_data_fin_template = pd.read_csv('html_concat_data_fin_template.csv')

# 3) Normalize chunk names (if needed) and map to colors
#      - Here we simply mirror 'Chunk' into 'normalized_chunk' (no renaming).
#      - Then assign colors via the manual map; missing keys get a default color.
html_concat_data_fin_template['normalized_chunk'] = html_concat_data_fin_template['Chunk']
html_concat_data_fin_template['color']                                          =
html_concat_data_fin_template['normalized_chunk'].map(manual_color_map)
```

```
# 4) Fallback color for unspecified chunks
#       - Use a neutral gray to visually de-emphasize non-mapped regions.
default_color = 'lightgray'
html_concat_data_fin_template['color'] = html_concat_data_fin_template['color'].fillna(default_color)

# 5) Initialize 3D canvas
fig = plt.figure(figsize=(8, 8))
ax = plt.subplot(1, 1, 1, projection='3d')

# 6) Scatter each chunk at its (X,Y, Z) with assigned color
#       - Marker size scales with Area (×2) to encode spatial weight.
for _, row in html_concat_data_fin_template.iterrows():
        ax.scatter(
                row['X'], row['Y'], row['Z'],
                c=row['color'],
                s=row['Area'] * 2
        )

# 7) Aesthetics for a clean, manuscript-friendly look
#       - Remove axis label text; hide tick labels but keep tick positions.
ax.set_xlabel('X', fontsize=0)
ax.set_ylabel('Y', fontsize=0)
ax.set_zlabel('Z', fontsize=0)

ax.set_xticklabels([])    # hide X tick labels
ax.set_yticklabels([])    # hide Y tick labels
ax.set_zticklabels([])    # hide Z tick labels

# Subtle 3D view and transparent panes
ax.view_init(20, 5)
ax.xaxis.set_pane_color((1.0, 1.0, 1.0, 0.0))
ax.yaxis.set_pane_color((1.0, 1.0, 1.0, 0.0))
ax.zaxis.set_pane_color((1.0, 1.0, 1.0, 0.0))

# 8) Final layout and render
plt.subplots_adjust(wspace=-0.15, hspace=-0.15)
plt.show()
```

Python code for Supplementary Figure 2

```
# -----------------------------------------------------------
# Purpose:
#     Compute pairwise Pearson correlations among baseline global features
#     with two-sided p-values, and visualize the correlation structure
#     using a heatmap. Significant cells (p < 0.05) are annotated with r.
#
# Study design:
#     - Input data: entire_final.csv
#     - Exclusion filter: Hemo1_Aneu2 == 0
#
# Feature set:
#     - Baseline arterial morphology features (Before_*)
#
# Statistics:
#     - Pearson correlation (two-sided) computed pairwise with pairwise deletion
#        of missing values (listwise within each pair).
#     - Upper triangle is computed and mirrored to enforce symmetry.
#
# Output:
#     - corr_matrix: symmetric matrix of Pearson r
#     - pvalue_matrix: corresponding two-sided p-values
#     - Heatmap (coolwarm, vmin=-1, vmax=1) with significant r values overlaid
# -----------------------------------------------------------

import pandas as pd
import numpy as np
import seaborn as sns
import matplotlib.pyplot as plt
from scipy.stats import pearsonr      # two-sided Pearson correlation

# 1) Load dataset and apply study filter
file_path = 'entire_final.csv'
df = pd.read_csv(file_path)
df = df[df['Hemo1_Aneu2'] == 0]      # apply exclusion criteria

# 2) Define arterial features and display labels (1:1 mapping, order preserved)
arterial_features = [
    'Before_MinMaxDiameterRatio', 'Before_LuminalCircularity', 'Before_Area',
    'Before_Perimeter', 'Before_MaxDiameter', 'Before_MinDiameter',
    'Before_MaxInscribedSphereR', 'Before_Curvature'
]
feature_labels = [
    'Minimum-maximum diameter ratio', 'Luminal circularity', 'Area', 'Perimeter',
    'Maximum diameter', 'Minimum diameter', 'Maximum inscribed sphere radius', 'Curvature'
]

# 3) Initialize result matrices (r and p) with proper indexing/column labels
n_features = len(arterial_features)
corr_matrix = pd.DataFrame(
    np.zeros((n_features, n_features)), columns=feature_labels, index=feature_labels
)
pvalue_matrix = pd.DataFrame(
    np.ones((n_features, n_features)), columns=feature_labels, index=feature_labels
)

# 4) Compute pairwise Pearson r and two-sided p-values
#       - Pairwise deletion: only rows with both features non-missing are kept
```

```
#       - Upper triangle (i <= j) is computed; values mirrored for symmetry
for i in range(n_features):
        for j in range(n_features):
                if i <= j:    # compute upper triangle including diagonal
                        feature_1 = arterial_features[i]
                        feature_2 = arterial_features[j]

                        x = df[feature_1]
                        y = df[feature_2]
                        mask = x.notna() & y.notna()
                        x_ = x[mask].astype(float)
                        y_ = y[mask].astype(float)

                        if len(x_) >= 2:                                        # need at least 2 paired observations
                                corr, pval = pearsonr(x_, y_)           # two-sided by default
                        else:
                                corr, pval = np.nan, np.nan

                        # Fill symmetric entries
                        corr_matrix.iloc[i, j] = corr
                        corr_matrix.iloc[j, i] = corr
                        pvalue_matrix.iloc[i, j] = pval
                        pvalue_matrix.iloc[j, i] = pval

# 5) Heatmap visualization (no annotations yet; color encodes r)
plt.figure(figsize=(9, 7))
ax = sns.heatmap(
        corr_matrix, cmap="coolwarm", vmin=-1, vmax=1,
        square=True, annot=False
)

# Cosmetic tweak: slightly thicker colorbar ticks
colorbar = ax.collections[0].colorbar
colorbar.ax.tick_params(width=1)

# Axis label formatting (diagonal x labels for readability)
plt.xticks(rotation=45, ha='right', fontsize=10)
plt.yticks(rotation=0, fontsize=10)

# 6) Overlay significant cells with numeric r (p < 0.05)
#       - Dynamic text color for contrast: white on |r| ≥ 0.5, else black
for i in range(corr_matrix.shape[0]):
        for j in range(corr_matrix.shape[1]):
                p = pvalue_matrix.iloc[i, j]
                if pd.notna(p) and p < 0.05:
                        value = corr_matrix.iloc[i, j]
                        text_color = 'black' if (pd.notna(value) and abs(value) < 0.5) else 'white'
                        ax.text(
                                j + 0.5, i + 0.5, f"{value:.2f}",
                                color=text_color, ha='center', va='center', fontsize=10
                        )

# 7) Title, layout, render
plt.title("Heatmap of Pearson Correlation Between Global Features\n", fontsize=14)
plt.tight_layout()
plt.show()
```

Python code for Supplementary Tables 6, 7, 8, 9 and 10

The following code exemplifies the analysis applied to the total group (n = 462) presented in Table S5. Subgroup-specific datasets were then constructed according to prespecified criteria, and the same analytical pipeline was applied to generate the remaining figure panels.

```
# ------------------------------------------------------------
# Purpose:
#     Run chunk-level Cox proportional hazards analyses for selected
#     morphological features (baseline and annualized change), and
#
# Study design:
#     - Input data: chunk_final.csv
#     - Exclusion filter: Hemo1_Aneu2 == 0 (analytic cohort)
#     - Time-to-event column: new_event_duration
#     - Event indicator: new_ischemic_stroke_event (1 = event, 0 = censored)
#     - Adjustment covariates: before_age, Sex
#
# Feature families:
#     - "Before_*"    → baseline values per chunk
#     - "*difference_per_year" → annualized longitudinal change per chunk
#     - Per the selection expression below, columns containing "length" are
#        excluded for "Before_*" features (operator precedence as written).
#
# Modeling details:
#     - For each feature column, fit a CoxPH model with the feature z-scored;
#        before_age and Sex are included unscaled as covariates.
#     - Complete-case analysis: rows with missing values are dropped
#        prior to fitting (model-wise N may vary).
#     - Results are retained for reporting if p < 0.10.
#
# Output:
#     - significant_df: raw per-feature Cox results (filtered by p < 0.10)
#     - result_df: formatted table with columns
#           * Arterial Features, Chunk, HR(95% CI), p-value
#        ordered by a predefined feature list and a predefined chunk list,
#        then displayed (Jupyter) and copied to clipboard.
#     - Numbers < 0.01 are formatted in scientific notation with Unicode
#        superscripts for readability.
# ------------------------------------------------------------

import pandas as pd
import numpy as np
from lifelines import CoxPHFitter


# 1) Load dataset and apply study filter
data = pd.read_csv('chunk_final.csv')
data = data[(data['Hemo1_Aneu2']==0)]
print(len(data))    # Sanity check: analytic cohort size after filtering

# 2) Analysis columns
duration_col = 'new_event_duration'                 # Time-to-event
event_col = 'new_ischemic_stroke_event'          # Event indicator (1 = event, 0 = censored)

# 3) Adjustment covariates
adjustment_vars = ['before_age', 'Sex']            # Prespecified covariates

# 4) Container for results
significant_results = []
```

```
# 5) Candidate features by name pattern (operator precedence as written)
columns_to_analyze = [col for col in data.columns if "difference_per_year" in col or "Before_" in col and "length" not in col]

# 6) Cox model instance
cox_model = CoxPHFitter()

# 7) Per-feature Cox modeling (feature z-scored; covariates unscaled)
for column in columns_to_analyze:
    if column not in [duration_col, event_col] + adjustment_vars:
        # Keep only analysis columns, drop missing rows (complete-case)
        df = data[[duration_col, event_col, column] + adjustment_vars].dropna()

        # Z-score transform for the feature column (std uses ddof=1)
        df[column] = (df[column] - df[column].mean()) / df[column].std(ddof=1)

        # Fit Cox PH model
        cox_model.fit(df, duration_col=duration_col, event_col=event_col)

        # Keep results if p < 0.10 (reporting threshold)
        p_value = cox_model.summary['p'].loc[column]
        if p_value < 0.1:
            hr = cox_model.summary['exp(coef)'].loc[column]
            # Map original column name to a human-readable label
            if "Before_" in column:
                column_name = "Baseline " + str(column).split("_")[2] + "_" + str(column).split("_")[1]
            elif "difference_per_year" in column:
                column_name = "Difference/year of " + str(column).split("_")[0]+ "_" + str(column).split("_")[1]
            ci_lower = cox_model.summary['exp(coef) lower 95%'].loc[column]
            ci_upper = cox_model.summary['exp(coef) upper 95%'].loc[column]
            significant_results.append({
                'Variable': column_name,
                'HR': hr,
                'CI Lower': ci_lower,
                'CI Upper': ci_upper,
                'p-value': p_value
            })

# 8) Assemble raw results
significant_df = pd.DataFrame(significant_results)

# 9) Display order for features and chunks (for consistent reporting)
feature_list = ["Baseline MinMaxDiameterRatio", "Baseline LuminalCircularity", "Difference/year of MinMaxDiameterRatio", "Difference/year of LuminalCircularity",
                "Baseline Area",  "Baseline Perimeter", "Baseline MaxDiameter", "Baseline MinDiameter", "Baseline MaxInscribedSphereR",
                "Difference/year of Area",  "Difference/year of Perimeter", "Difference/year of MaxDiameter", "Difference/year of MinDiameter", "Difference/year of MaxInscribedSphereR",
                "Baseline Curvature", "Difference/year of Curvature"]
chunk_list = ["RtICA", "LtICA", "RtBasalMCA", "LtBasalMCA", "RtBasalACA", "LtBasalACA", "RtPialMCA", "LtPialMCA", "RtPialACA", "LtPialACA",
              "RtVA", "LtVA",  "BA", "RtCbll", "LtCbll", "RtBasalPCA", "LtBasalPCA", "RtPialPCA", "LtPialPCA", "ACoA"]

# 10) Unicode superscript helper for scientific notation
def format_superscript(num):
```

```
    superscript_map = {
        '0': '⁰', '1': '¹', '2': '²', '3': '³', '4': '⁴',
        '5': '⁵', '6': '⁶', '7': '⁷', '8': '⁸', '9': '⁹', '-': '⁻'
    }
    return ''.join(superscript_map.get(char, char) for char in str(num))

# 11) Build formatted table entries (HR, 95% CI, p-value)
table_data = []
for index, row in significant_df.iterrows():
    variable = row['Variable']   # Combined label used below to infer feature/chunk
    parts = variable.split('_')
    feature = next((f for f in feature_list if f in parts), 'Unknown')
    chunk = next((c for c in chunk_list if c in parts), 'Unknown')
    hr = row['HR']
    ci_lower = row['CI Lower']
    ci_upper = row['CI Upper']
    p_val = row['p-value']

    # Scientific notation with superscripts for very small numbers
    if hr < 0.01:
        hr_base, hr_exp = f"{hr:.2e}".split('e')
        hr_exp = format_superscript(int(hr_exp))
        hr_text = f"{hr_base} × 10{hr_exp}"
    else:
        hr_text = f"{hr:.2f}"

    if ci_lower < 0.01:
        ci_lower_base, ci_lower_exp = f"{ci_lower:.2e}".split('e')
        ci_lower_exp = format_superscript(int(ci_lower_exp))
        ci_lower_text = f"{ci_lower_base} × 10{ci_lower_exp}"
    else:
        ci_lower_text = f"{ci_lower:.2f}"

    if ci_upper < 0.01:
        ci_upper_base, ci_upper_exp = f"{ci_upper:.2e}".split('e')
        ci_upper_exp = format_superscript(int(ci_upper_exp))
        ci_upper_text = f"{ci_upper_base} × 10{ci_upper_exp}"
    else:
        ci_upper_text = f"{ci_upper:.2f}"

    # p-value formatting
    if p_val < 0.001:
        p_value_text = "<0.001"
    else:
        p_value_text = f"{p_val:.3f}"

    # Collect final row
    table_data.append({
        'Arterial Features': feature,
        'Chunk' : chunk,
        'HR(95% CI)': f'{hr_text} ({ci_lower_text} - {ci_upper_text})',
        'p-value': p_value_text
    })

# 12) Final table and ordering
result_df = pd.DataFrame(table_data)

# Order by predefined feature list, then chunk list
result_df['Feature Order'] = result_df['Arterial Features'].map({feature: i for i, feature in
```

```
enumerate(feature_list)})
result_df['Chunk Order'] = result_df['Chunk'].map({chunk: i for i, chunk in enumerate(chunk_list)})

result_df = result_df.sort_values(['Feature Order', 'Chunk Order']).drop(['Feature Order', 'Chunk Order'], axis=1).reset_index(drop=True)

# 13) Display (Jupyter) and copy to clipboard
styled_table = result_df.style.set_table_attributes('style="width:80%"') \
    .set_properties(**{'background-color': 'white', 'color': 'black', 'text-align': 'left'}) \
    .set_table_styles([{'selector': 'th', 'props': [('text-align', 'left'), ('background-color', 'white')]}])

from IPython.display import display
display(styled_table)

# Copy to clipboard for convenient transfer to a manuscript table
result_df.to_clipboard(index=False)
```
